# Roles of human colonic bacteria in pectin utilization and associated cross-feeding networks revealed using synthetic co-cultures

**DOI:** 10.1099/mic.0.001559

**Published:** 2025-05-29

**Authors:** Michael Solvang, Freda M. Farquharson, Graham Horgan, Sushila Pisano, Jesper Holck, Birgitte Zeuner, Wendy R. Russell, Petra Louis

**Affiliations:** 1 Rowett Institute, University of Aberdeen, Foresterhill, Aberdeen AB25 2ZD, UK; 2 Biomathematics and Statistics Scotland, Aberdeen, UK; 3 Department of Biotechnology and Biomedicine, Technical University of Denmark, Søltofts Plads 2800 Kgs. Lyngby, Denmark

**Keywords:** carbohydrate metabolism, cross-feeding, dietary fibre, human gut microbiota, pectin, synthetic co-culture

## Abstract

Dietary fibre is a crucial component of healthy diets via its action on the human gut microbiota, but fibre intake is well below current international dietary guidelines at the population level. Pectin is a fibre constituent in fruit and vegetables that has the promise to promote a healthy and diverse microbiota. It is a highly complex molecule, and its composition differs between plants. Here, we assessed the ability of a panel of 23 human gut bacteria to ferment pectins extracted from different plants based on their genome carriage of carbohydrate-active enzymes (CAZymes) and their growth in pure culture on several mono-, oligo- and polysaccharides, as well as pectins from different plant sources. Growth behaviour was overall in good agreement with CAZyme carriage, and the results were used to design synthetic co-culture communities with different combinations of pectin degraders, pectin cross-feeders and background strains not expected to play a major role in pectin degradation. For pectin degraders, *Lachnospira eligens* DSM 3376 outcompeted *Phocaeicola vulgatus* DSM 1447 and *Segatella copri* DSM 18205*,* which appeared to act more as a cross-feeder in the presence of *L. eligens* DSM 3376. Between the cross-feeders, *Roseburia intestinalis* M50/1 likely utilized breakdown products from the pectin backbone and side chains, whereas *Faecalibacterium duncaniae* A2-165 grew better in co-culture on homogalacturonan-rich pectins. Our work will help to explain individual-specific responses to pectin intake based on microbiota compositional variation and contribute to the design of personalized dietary strategies to optimize the microbiota.

## Graphical Abstract

Combining genome analysis, pure culture and synthetic co-culture growth behaviour on a range of pectin-related substrates to reveal microbial interactions of human gut microbes in pectin fermentation.

## Data Availability

The processed data that support the findings of this study are available in the supplementary material of this article, and raw data not provided are available from the corresponding author upon request.

## Introduction

Plant-based dietary fibre plays an important role in the maintenance of human health as a major nutrient source for the microbiota in the human large intestine [1,2]. Pectin is the structurally most complex fibre component in the plant cell wall, consisting of four domains: homogalacturonan (HG), rhamnogalacturonan I and II (RG-I and RG-II) and xylogalacturonan. Quantitatively, HG and RG-I account for ~90% of the pectin molecule [3] and are consequently the domains that likely have the largest impact on the gut microbiota. Pectin composition varies from plant to plant, by the ratio between the different domains, the ratio of the side chain to backbone residues in the RG-I domain (i.e. degree of branching), the ratio of arabinose to galactose in the side chains of the RG-I domain and the degree of methylation [3]. To degrade the complex pectin molecule, gut bacteria require a large repertoire of carbohydrate-active enzymes (CAZymes) [4]. Carbohydrate esterases (CEs) remove methyl or acetyl groups from the pectin backbone, and certain polysaccharide lyase (PL) and glycoside hydrolase (GH) families cleave the pectin backbone (Table 1). As HG constitutes large parts of the pectin molecule (usually over 50%, [3]) and only a select few PLs and GHs can break down the HG domain, those enzymes serve as important diagnostic indicators for pectin degradation. The degradation of the RG-I side chains involves CAZymes not specific to pectin, as they may also degrade other cell wall polysaccharides, whereas the degradation of RG-II involves both pectin-specific and non-pectin-specific CAZymes [5].

**Table 1.** Number of CAZymes required for the degradation of the two major pectin domains, HG and RG-I, predicted in the genomes of the bacteria examined in this study*

­ 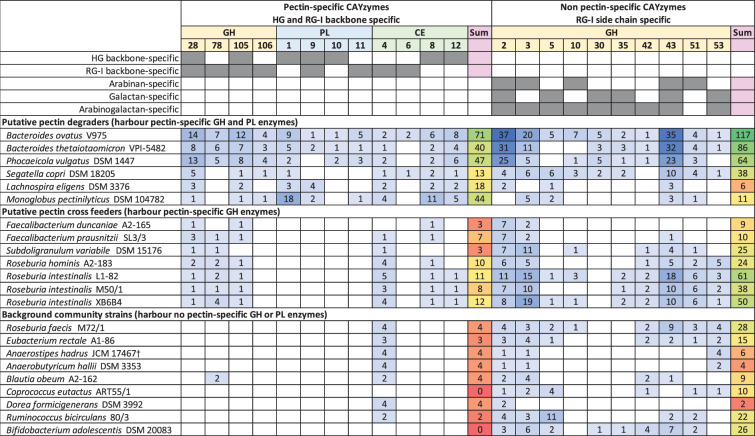

*Adapted from Hamaker and Tuncil [16] with additional information from http://www.cazy.org.

†*Anaerostipes hadrus* type strain JCM 17467 information is given here, as *Anaerostipes hadrus* SSC/2, used in this study, was not available in the CAZy database.

The breakdown and utilization of pectins by the human gut microbiota are dependent on the activity of primary pectin degraders, which include *Bacteroides* spp. [6] and a small number of *Bacillota*, including *Lachnospira* (formerly *Eubacterium*) *eligens* and *Monoglobus pectinilyticus* [7,8]. Some strains of *Faecalibacterium* have also been reported to show limited growth on pectin; however, this appeared largely to be due to the utilization of pectic oligosaccharides rather than intact pectin [7,9]. These bacteria may therefore be cross-feeders that utilize the intermediate breakdown products released by primary pectin degraders, referred to here as pectin cross-feeding. Pectin degradation might also stimulate a second type of cross-feeding (metabolite cross-feeding), where fermentation products such as lactate can be utilized [1]. Faecal incubation experiments with pectin indicate that bacteria can be differentially stimulated based on the structural features of the pectin used. Larsen *et al*. investigated *in vitro* faecal fermentation of pectin from orange, lemon and sugar beet, extracted with both mild and harsh methods [10]. They showed that the extraction method affected the structural characteristics of the pectin, which in turn affected bacterial growth. For example, arabinose-rich pectin correlated with the relative abundance of *Segatella* (formerly *Prevotella) copri*, whereas galacturonic acid-rich pectin correlated with the relative abundance of *Faecalibacterium prausnitzii*. Solvang *et al*. extracted plant cell wall material and pectins from different plants and found that compositional differences between pectins correlated with which bacteria were differentially abundant after *in vitro* human faecal incubation, with arabinan-rich beetroot and beet leaf pectin stimulating the *Bacteroides* genus and galactan-rich carrot pectin the *Eubacterium rectale* group and *Fusicatenibacter* [11].

The microbial interactions during pectin fermentation in the intestine, such as substrate competition and cross-feeding interactions, are not well understood but likely play a major role in the microbiota response to dietary pectin in individuals with varying microbiota composition. Studies using faecal microbiota provide valuable insights, but due to their complexity and differing composition between individuals and over time, it is difficult to obtain mechanistic insights into microbial interactions during fibre metabolism. Synthetic co-cultures consisting of several microbes are increasingly being utilized as models to assess microbial community interactions [12]. While they are obviously much simpler than the gut microbiota, they enable the assessment of microbial competition and cooperation between individual microbes that occupy similar ecological niches in a defined and reproducible setting. Here, we assessed CAZyme carriage and pure culture growth kinetics of 23 gut bacteria occupying different ecological niches (primary pectin degraders, pectin and metabolite cross-feeders and background strains assumed not to play a major role in pectin breakdown) to establish their metabolic potential on different pectin-related substrates, including major pectin monosaccharides, RG-I side chain carbohydrates, oligo-galacturonans and intact pectins from several plants. We then set up synthetic co-cultures with a selection of strains to assess their community behaviour.

## Methods

### Pectin extraction and monosaccharide composition analysis

Pectin was extracted from five different plants, organically farmed apples (*Malus domestica* variety Fiesta), beet leaves (*Beta vulgaris* variety Robuschka), beetroots (*Beta vulgaris* variety Robuschka), carrots (*Daucus carota* variety Rodelika) and kale (*Brassica oleracea* variety sabellica). Plants were bought from Vital Veg (Midmar, Aberdeenshire, UK) and processed within 3 days of purchasing as detailed previously [11]. Briefly, the fresh produce was freeze dried and freezer milled in liquid nitrogen, and alcohol-insoluble residue (AIR) was prepared. Pectin was extracted on two different occasions (pectins I and II) from AIR, and both preparations had very similar monosaccharide composition [11]. Pectin II was used for pure culture experiments E1 and E2, and pectin I was used for pure culture experiments E3 and E4 and the co-culture experiment.

### Bacterial strains and growth conditions

All strains were obtained either from the Rowett strain collection or the German Collection of Microorganisms and Cell Cultures (DSMZ) (Table S1, available in the online Supplementary Material). Pure culture growth tests were performed over five independent experiments (E1–E5; for an overview, see Fig. S1), and co-culture studies were performed in a single experiment using the same medium batch and pre-cultures. Bacteria were pre-cultured anaerobically (under carbon dioxide) in Hungate tubes in 7.5 ml modified yeast extract casitone fatty acids (YCFA) medium [13] overnight at 37 °C. The carbon source was either glucose (pure culture experiments E1–2 and E4–5, all strains in the co-culture experiment, designated glc), glucose plus HG (pure culture experiment E3) or commercial apple pectin (Sigma Aldrich 76282; pectin degraders in the co-culture experiment, designated pec), all at 0.2% w/v. The OD (650 nm; Novaspec II visible spectrophotometer, Pharmacia LKB Biotechnology AB, Uppsala, Sweden) of each pre-culture was measured twice 30 min apart to assess the growth stage of the culture (Tables S2 and S3). For the preparation of co-culture inocula, pre-culture aliquots were diluted to OD <0.4 with basal YCFA medium (no added substrate) to ensure they were in the linear range for OD measurements. Culture volumes representing 0.05 OD equivalents for each strain were combined and topped up to 5 ml with basal YCFA medium. For molecular community analysis at *t*=0 h, 1 ml community inoculum was centrifuged (10,000 ***g*** for 10 min at 4 °C; Jouan 22, DJB Labcare Ltd, Newport Pagnell, UK). The supernatant was removed, and the cell pellet was resuspended in 122 µl of MT buffer (FastDNA spin kit for soil, MP Biomedicals, Illkirch, France) and stored at −70 °C until DNA extraction.

Pure and co-culture fermentation experiments were conducted in modified YCFA medium in microtitre plates (Costar 3370 plates, Corning Inc., ME, USA) as described before [14] in a Whitley A135 HEPA anaerobic workstation (Don Whitley, Shipley, UK) under an atmosphere of 80% N_2_, 10% CO_2_ and 10% H_2_. The growth media were prepared with boiling under carbon dioxide as for Hungate tubes, and the pH was adjusted at ~65 °C to 6.1 for a target pH of ~6.3–6.5 after equilibration in the anaerobic cabinet for at least 48 h before inoculation (Tables S3 and S4). The following carbon sources were used for pure culture (0.2% w/v) and co-culture incubations (0.5% w/v): d-(+)-glucose (Fisher Scientific G/0500/53); l-(+)-arabinose (Sigma Aldrich A3256); d-(+)-galactose (Sigma Aldrich G0750); d-galacturonic acid (Sigma Aldrich 73960); d-(+)-mannose (Sigma Aldrich M8296); l-rhamnose (Sigma Aldrich R3875); d-(+)-xylose (Sigma Aldrich X1500); apple pectin (Sigma Aldrich 76282); citrus pectin (Sigma Aldrich P9135); arabinogalactan (larch wood, Sigma Aldrich A-2012); arabinan (sugar beet, Megazyme, P-ARAB); HG (citrus pectin Megazyme P-PGACIT); rhamnogalacturonan (Megazyme P-RHAM1); galactan (potato, Megazyme P-GALPOT); oligo-galacturonans with degree of polymerization (DP) of 4, 5, 6 and 8 (prepared as described previously [7,15]); in-house prepared pectins from apple, beet leaf, beetroot, carrot and kale [11]; and a pectin monosaccharide mix consisting of the four major pectin constituents (65% d-galacturonic acid, 20% d-galactose, 10.5% l-arabinose and 3.5% l-rhamnose; based on carrot pectin preparation I [11]). Media containing monosaccharide substrates were autoclaved (pure culture experiments E1–2 and co-culture experiment). For all other substrates and monosaccharides for experiments E3–5, the basal medium was prepared by reducing the amount of water by 33.3% and autoclaved. Each substrate was sterilized by vortexing in absolute ethanol and drying in vacuo. The sterile substrates were dissolved in the appropriate volume of water under flushing CO_2_ and added to the medium directly before the incubation. Growth differences were observed for a few strains on glucose between different experiments; however, no clear trend between E1–2 and E3–5 data was apparent that would suggest that the different sterilization methods for monosaccharides did have a major impact on growth (Fig. S1). Basal medium without added carbon source (no CHO (carbohydrate)) served as a negative control to establish growth on other medium components.

Plate wells containing 198 µl of medium were inoculated with 2 µl of pure culture or co-culture in triplicate. The inoculated plates were sealed with Q-optical seals (Bio-Rad, Watford, UK) and placed in a plate stacker robot (BioStack 4, microplate stacker, BioTek, Swindon, UK) with plates containing 200 µl water per well at the top and bottom to minimize condensation in the growth plates. OD (650 nm) was measured every 20 min for up to 48 h (EPOCH|^2^ microplate reader, BioTek) after mixing by shaking [double orbital mode, frequency: 425 cpm (3 mm)]. After subtracting blank medium OD values, growth was expressed as maximum OD (OD_max_). For pure cultures, no CHO OD_max_ was subtracted from OD_max_ of substrate-containing cultures (net OD_max_). For strains and substrates with an average net OD_max_ >0.1, exponential phase growth rates were determined from blank-subtracted OD data (OD data, net OD_max_, growth rates and parameters for growth rate calculations are provided in Table S4). Data were not compared statistically as there was variation for some strains and substrates between experiments (Fig. S1), likely due to small differences in medium batches.

At the end of the growth experiment, microbial community growth plates were removed from the anaerobic cabinet and placed on ice. The cultures were transferred to a segmented 96-well plate (Axygen PCR-96-SG-C; Flintshire, UK), sealed (2239444 plate seals; Bio-Rad) and centrifuged (4,000 ***g***; 15 min; 4 °C; ALC PK 131R, DJB Labcare Ltd). The supernatant was removed, and cell pellets were resuspended in 122 µl of MT buffer (FastDNA spin kit for soil, MP Biomedicals). The suspension was transferred into a Lysis Matrix E tube containing 978 µl sodium phosphate buffer (FastDNA spin kit for soil, MP Biomedicals) and mixed before being stored at −70 °C until DNA extraction.

### Analysis of CAZyme carriage

CAZymes believed to be involved in the degradation of different pectin components were identified based on the information given by Hamaker and Tuncil [16] and on the CAZy database (https://www.cazy.org/ [4]). CAZyme carriage of different bacterial strains was obtained from the CAZy database in March 2025. Details on the genome information accessed are given in Table S1.

### Molecular community analysis of synthetic co-cultures

Microbial community DNA was extracted with the FastDNA spin kit for soil (MP Biomedicals) according to the manufacturer’s instructions. DNA concentrations were measured using a Qubit 2.0 Fluorometer (Thermo Fisher Scientific, Paisley, UK). Molecular community analysis was conducted by multiplex PCR as described previously [14]. Strain-specific primers (Table S5) were designed in single-copy genes using NCBI Primer-blast (https://www.ncbi.nlm.nih.gov/tools/primer-blast/ [17]) containing universal tag sequences at the 5′ ends to allow amplification with universal primers and incorporation of fluorescent label cyanine 5. An internal standard (genomic DNA from *Corynebacterium glutamicum* DSM 1412 (Cg); DSMZ, Braunschweig, Germany) was added to the community DNA. The multiplex PCRs were set up with 1× Qiagen Multiplex PCR kit reagent (Qiagen, Manchester, UK) in PCR plates (Starlab, Milton Keynes, UK) as follows: 5 ng sample DNA, 1.25 ng of Cg, 0.02 µM of each strain-specific primer and 2 µM of each universal tag primer (Table S5) in a total volume of 20 µl. A response factor for each individual strain relative to the Cg internal standard was generated by setting up PCR reactions with equal amounts of DNA (2.5 ng/20 µl) for the pure strains and the Cg internal standard. Plates were sealed with a plate seal (Bio-Rad, Hemel Hempstead, UK). The PCR cycling conditions were as follows: 1 cycle of 95 °C for 15 min; 18 cycles of 94 °C for 30 s, 57 °C for 90 s and 72 °C for 60 s; and 1 cycle of 72 °C for 10 min ending with a 10 °C holding temperature. Fragment analysis was carried out by capillary electrophoresis on a Beckman Coulter CEQ 8000 GeXP Genetic Analysis system (Beckman Coulter, High Wycombe, UK). An aliquot of PCR product was diluted 10× in molecular biology water, and 2 µl was added to an Axygen PCR-96-FS-C plate (Axygen, Flintshire, UK), with each well containing a master mix consisting of CEQ size standard 400 (0.35 µl/well; AB Sciex, Warrington, UK) and sample loading solution (30 µl/well; AB Sciex). The plates were mixed, and one drop of mineral oil was added (AB Sciex) and analysed under the following conditions: capillaries at 50 °C, denaturation at 90 °C for 120 s, injection at 2 kV for 30 s and separation at 6 kV for 40 min.

The raw data were processed and filtered using the GenomeLab GeXP software with the Peak Criteria as follows: slope threshold=2 and relative peak height threshold=2%. Data were quality checked to ensure that unexpected and/or comigrating peaks were absent. Peaks with relative fluorescence units (RFUs) below 150 were excluded, and peak heights were used for downstream data analysis. Response factors for the individual strains were calculated from the PCR reactions containing equal amounts of strain DNA and Cg internal standard (strain RFU/Cg RFU). The strain-specific peaks were normalized against the Cg peak, the corresponding response factor was applied [strain RFU/(Cg RFU × strain response factor)], and relative abundance calculated from the sum of all adjusted strain peak heights. Note that this system is less sensitive than quantitative PCR or amplicon sequencing for microbial community analysis, and low abundance strains may not be detected.

### Statistical analysis

Comparisons of maximum and final ODs between co-cultures were done using an independent sample t-test, and one-way ANOVA was conducted in SPSS 25.0 (IBM, Chicago, USA), and a *P*-value≤0.05 was considered statistically significant for all analyses. In the few cases where the normality of the data was not demonstrable, the data were log transformed (base 10) to obtain normality. For ANOVA, comparison between treatment groups and adjustment for multiple testing were done with Tukey’s post hoc tests, where the assumption of homogeneity of variances was met, and the Games–Howell post hoc tests were done where homogeneity of variance was not met.

Analysis of the co-culture community composition was conducted by three-way ANOVA (linear model) since three factors potentially affected the final composition (pre-culture substrates of the pectin degraders, growth substrates and inoculum composition) using R 3.6 (R Foundation for Statistical Computing, Vienna, Austria). Comparison between treatment groups and adjustment for multiple testing were done with Tukey’s post hoc test. The analysis was conducted on both the original values and on log-transformed values. Residuals from the original scale model were plotted and examined, and when substantially skewed distributions were apparent, the significance of factors was taken from the log scale version of the analysis when this differed from the original scale version. A *P*-value≤0.05 was considered statistically significant for all analyses. The Bray–Curtis dissimilarity was used [18] to assess beta-diversity.

## Results

### Pectin-related CAZyme profiles of selected strains

A total of 22 bacterial strains from 3 different phyla were selected for assessment of genomic carriage of CAZymes involved in pectin degradation (Table 1). They represent species commonly found in the gut microbiota of healthy humans [19] and species or genera for which pectin or oligo-galacturonan utilization has been demonstrated by culture or hypothesized from genome information (Table S1). Pectin-specific CAZyme genes (involved in either HG- or RG-I-backbone cleavage) belonging to all three enzyme classes (GH, PL and CE) were found in all *Bacteroidota* species, *Monoglobus pectinilyticus* DSM 104782 and *L. eligens* DSM 3376, suggesting that they are primary pectin degraders (Table 1). *Bacteroidaceae* species carried the highest number of genes, in accordance with them being generalists and carrying high CAZyme numbers in their genomes [1]. *Monoglobus pectinilyticus* DSM 104782 also carried a high number of pectin-specific CAZymes; however, this was mainly driven by CAZyme families PL1 and CE8, suggesting a high degree of specialization. The two *Faecalibacterium* strains, *Subdoligranulum variabile* DSM 15176, *Roseburia hominis* A2-183 and all *Roseburia intestinalis* strains contained pectin-specific GH and CE, but not PL, CAZymes, which suggests that they can utilize galacturonic acid-containing pectin backbone constituents liberated by pectin primary degraders. *Blautia obeum* A2-162 contained two GH78 and two CE4 genes, indicating that it may be able to cross-feed on RG-I, but not HG, breakdown products (Table 1).

For CAZymes potentially involved in RG-I side chain degradation but not specific to only pectins (Table 1), *Bacteroidaceae* species carried the highest number, followed by *Roseburia intestinalis* strains. The carriage in other strains examined here varied widely, suggesting large differences in their capacity to utilize pectin side chain components. Strains that did not harbour HG backbone-specific GH or PL enzymes were designated background strains, but they may still be involved in the utilization of pectin side chain carbohydrates or receive pectin constituents cross-fed by other bacteria. Two known lactate cross-feeders, *Anaerostipes hadrus* SSC/2 [20] and *Anaerobutyricum* (formerly *Eubacterium*) *hallii* DSM 3533 [21] only harboured few CAZymes (Table 1), suggesting that any benefits they may gain from community pectin breakdown would likely be due to lactate rather than carbohydrate cross-feeding.

### Pure culture growth

#### Experimental setup and general growth characteristics

All strains were tested for their ability to grow on different monosaccharides, pectin constituents and pectins. *Monoglobus pectinilyticus* DSM 104782 grew very poorly under all conditions and had to be excluded. However, a second *L. eligens* strain available, I42, was included in the growth tests. Maximum growth on glucose (OD_max_) tended to be lower in the growth experiments (performed in 96-well plates) than in the Hungate tube-grown pre-cultures, particularly for the *Bacteroidota* and a few *Bacillota* strains (Table S2), presumably due to the plate reader conditions or gas atmosphere in the anaerobic cabinet. Some variation in OD_max_ or lag phase was also seen between independent experiments (Fig. S1 and Table S4); therefore, growth characteristics were not compared statistically between strains or substrates, as the main objective was to establish the principal ability of strains to grow on different substrates. Net maximum OD (net OD_max_, OD of no CHO control subtracted) of ≥0.1 was regarded as positive growth on the respective substrate (Table 2).

**Table 2.** Average net OD_max_ (no CHO negative control subtracted) of pure cultures of 22 bacterial strains grown on different substrates*

­ 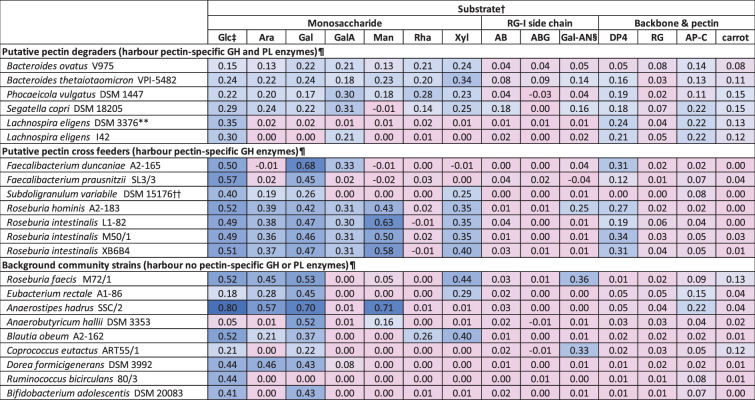

*sd, number of replicates and pre-culture ODs are given in Table S2.

†Substrate abbreviations; monosaccharides: Ara, arabinose; Gal, galactose; GalA, galacturonic acid; Glc, glucose; Man, mannose; Rha, rhamnose; Xyl, xylose; RG-I side chains: AB, arabinan; ABG, arabinogalactan; Gal-AN, galactan; backbone and pectins: AP-C, commercial apple pectin; carrot: in-house carrot pectin; DP4, oligo-galacturonan with four monomeric units; RG, rhamnogalacturonan.

‡Pre-culture substrate (experiment E3 also contained HG in addition to glucose).

§Growth on galactan was not consistent for *Faecalibacterium* strains, *Roseburia intestinalis* M50/1 and *Roseburia intestinalis* XB6B4 (see Table S4 and Fig. S1). As no clear pattern of contamination could be established and other data were in accordance with current knowledge for the strains and substrates, replicates that showed growth for those strains were excluded.

¶Strains were assigned based on CAZyme carriage as per Table 1.

**Absence of growth of *L. eligens* DSM 3376 on galacturonic acid was observed in three independent growth experiments, including E3 where HG was added to pre-cultures to assess if substrate adaptation was required (Fig. S1 and Table S4).

††*Subdoligranulum variabile* DSM 15176 did not show growth on either GalA or DP4 and was therefore not regarded as a pectin cross-feeder for further work in this study.

#### Growth of all strains on monosaccharides

With regard to the putative pectin degraders, the four *Bacteroidota* strains exhibited growth on all monosaccharides, except *Segatella copri* DSM 18205 on mannose (not present in pectins but indicative of growth on hemicellulosic fibres). The two *L. eligens* strains grew only on glucose and exhibited divergent behaviour on galacturonic acid, with only *L. eligens* I42 showing growth (based on three independent experiments, Table S4 and Fig. S1). For pectin cross-feeders, the two *Faecalibacterium* strains grew well on glucose and galactose, but only *Faecalibacterium duncaniae* A2-165 was able to grow on galacturonic acid (based on two independent experiments, Table S4 and Fig. S1). *Subdoligranulum variabile* DSM 15176 grew on four of the monosaccharides but not galacturonic acid, whereas the *Roseburia hominis* and *Roseburia intestinalis* strains grew on all monosaccharides apart from rhamnose. The growth profiles of background strains varied; however, galactose was a major substrate for most strains, whereas none of the strains was able to grow on galacturonic acid (Table 2).

#### Growth of all strains on RG-I side chains and selected pectins

None of the 22 strains tested exhibited growth (net OD_max_ ≥0.1) on arabinogalactan or rhamnogalacturonan, and only *Segatella copri* DSM 18205 grew on arabinan (Table 2). Several strains showed growth on galactan, but this was not always consistent across different experiments for a few strains (Table S4 and Fig. S1). For the two pectins (commercial apple pectin and in-house carrot pectin) tested with all strains, all putative pectin degraders showed growth on at least one pectin, and most also grew on DP4 oligo-galacturonan. None of the pectin cross-feeders could grow on pectins, but all apart from *Subdoligranulum variabile* DSM 15176 grew on DP4 oligo-galacturonan. None of the background community strains were able to grow on DP4 oligo-galacturonan, and most of those strains also could not grow on pectins. The limited growth of *E. rectale* A1-86 and *Anaerostipes hadrus* SSC/2 on commercial apple pectin and *Roseburia faecis* M72/1 and *Coprococcus eutactus* ART55/1 on carrot pectin (Table 2) may reflect the utilization of pectin side chains.

#### Growth of pectin degraders and cross-feeders on oligo-galacturonans and further pectins

**Fig. 1.**
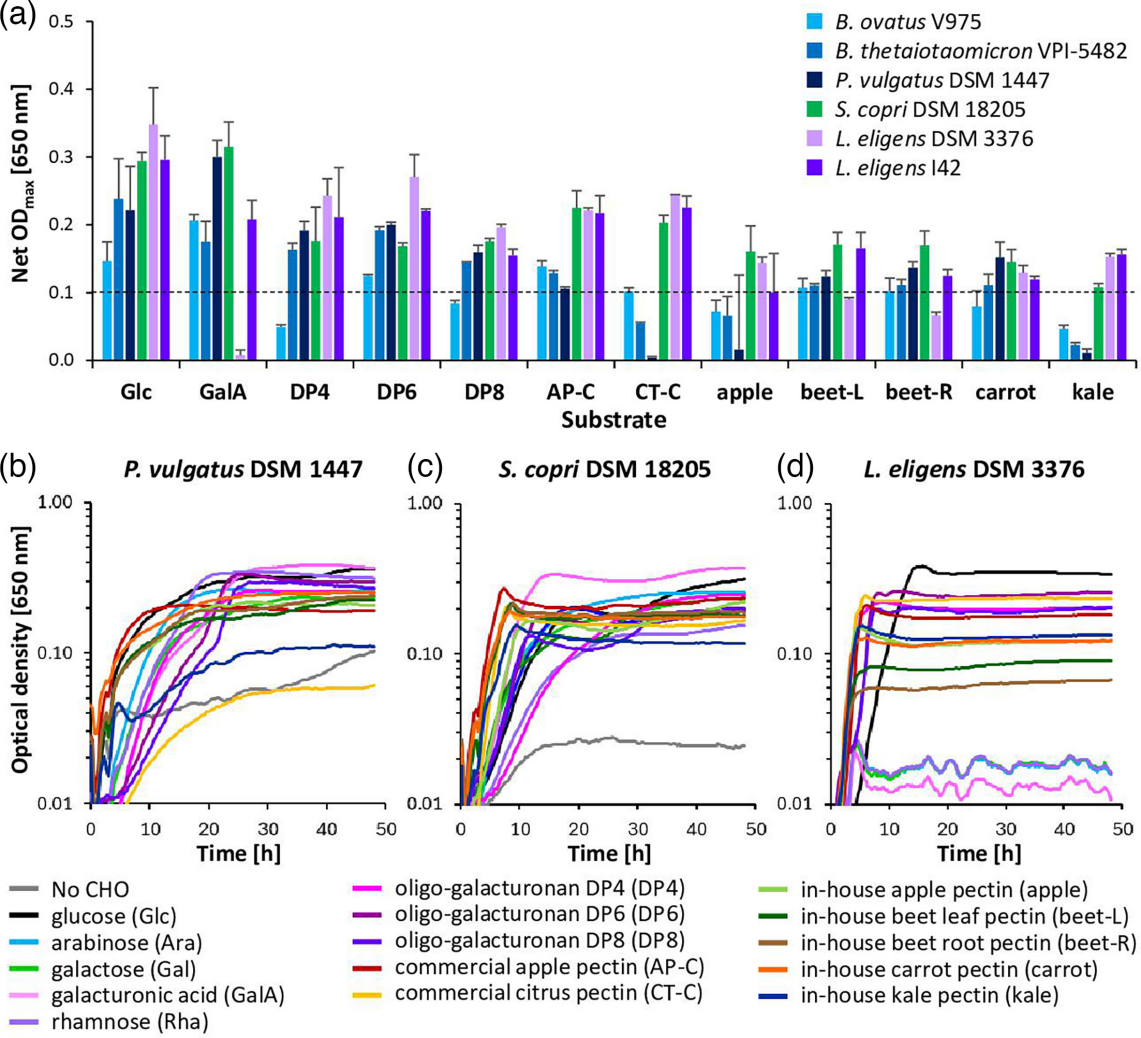
Pure culture growth of pectin degraders. (a) Net maximum growth (net OD_max_, no CHO subtracted) of six pectin degraders on glucose (Glc); galacturonic acid (GalA); DP4, DP6 and DP8 oligo-galacturonan; commercial apple (AP-C) and citrus (CT-C) pectin; and in-house apple, beet leaf (beet-L), beetroot (beet-R), carrot and kale pectin; net OD_max_ ≥0.1 (dashed line) regarded as positive growth. (b–d) Growth curves (average of triplicates from one experiment) of three pectin degraders selected for the co-culture experiment, *Phocaeicola vulgatus* DSM 1447, *Segatella copri* DSM 18205 and *L. eligens* DSM 3376, on co-culture-relevant substrates. Growth curves for all strains on all substrates are given in Fig. S1, underlying growth data are given in Table S4 and maximum growth rates are provided in Tables 3 and S6.

**Fig. 2.**
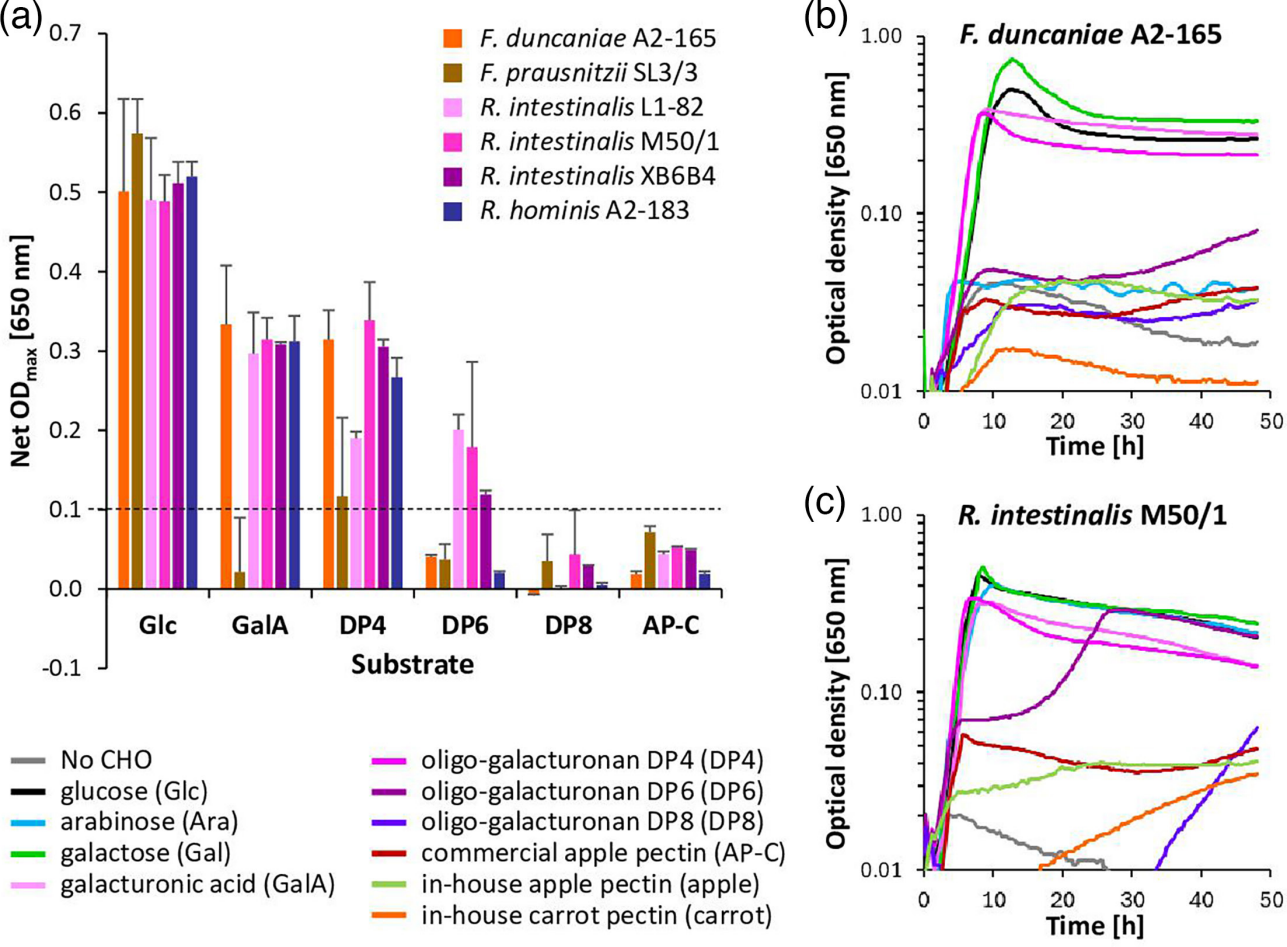
Pure culture growth of pectin cross-feeders. (a) Net maximum growth (net OD_max_, no CHO subtracted) of six cross-feeders on glucose (Glc); galacturonic acid (GalA); DP4, DP6 and DP8 oligo-galacturonan; and commercial apple (AP-C) pectin; net OD_max_ ≥0.1 (dashed line) regarded as positive growth. (b, c) Growth curves (average of triplicates from one experiment) of two pectin cross-feeders selected for the co-culture experiment, *Faecalibacterium duncaniae* A2-165 and *Roseburia intestinalis* M50/1, on co-culture-relevant substrates. Growth curves for all strains on all substrates are given in Fig. S1, underlying growth data are given in Table S4 and maximum growth rates are provided in Tables 3 and S6.

Pectin degraders and cross-feeders were further investigated for their growth on DP6 and DP8 oligo-galacturonans. *S. variable* DSM 15176 was excluded, as it was neither able to grow on galacturonic acid nor DP4 oligo-galacturonan. *Bacteroides ovatus* V975 grew poorly on oligo-galacturonans (only DP6 net OD_max_ ≥0.1), but all other pectin degraders grew on all oligo-galacturonans (Fig. 1). The pectin cross-feeders did not grow well on DP6 or DP8, but limited growth (mainly for DP6) was found after an extensive lag phase for the *Roseburia intestinalis* strains, with some variation between strains and experiments (Figs 2 and S1). Pectin degraders were also grown on further pectins. *Segatella copri* DSM 18205 and *L. eligens* I42 grew on all pectins, whereas *L. eligens* DSM 3376 showed less growth on beet leaf and beetroot pectin. The *Bacteroidaceae* strains mostly grew less well on commercial citrus pectin and in-house apple and kale pectin than on the other pectins (Figs 1 and S1). Growth on HG was also tested for pectin degraders and cross-feeders. Due to the viscous nature of the substrate, many growth curves were not smooth, and net OD_max_ could not be determined reliably; however, growth was consistently only observed for pectin degraders (Fig. S1).

**Table 3.** Average maximum growth rate for strain-substrate combinations with an average net OD_max_ ≥0.1 on growth substrates used for co-culture experiments*

­ 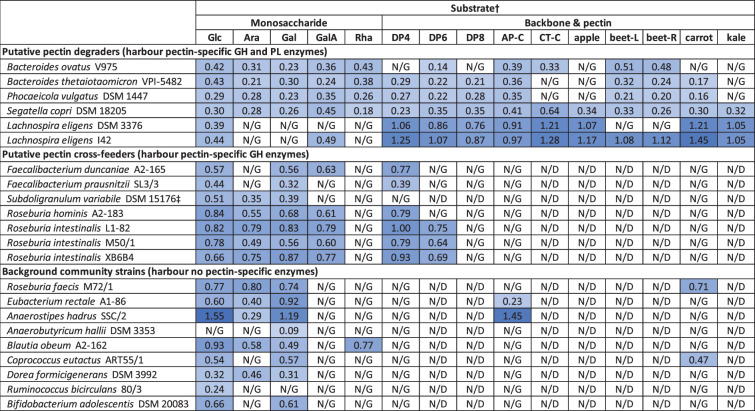

*Growth rate data for all substrates, together with sd and number of replicates, are given in Table S6.

†Abbreviations; substrates, monosaccharides: Ara, arabinose; Gal, galactose; GalA, galacturonic acid; Glc, glucose; Rha, rhamnose; backbone and pectins: AP-C, commercial apple pectin; apple, in-house apple pectin; beet-L, in-house beet leave pectin; beet-R, in-house beetroot pectin; carrot: in-house carrot pectin; CT-C, commercial citrus pectin; DP4/6/8, oligo-galacturonan with four/six/eight monomeric units; kale, in-house kale pectin. Data labels: N/D, not determined; N/G, net OD_max_<0.1 as per Table S2.

‡*Subdoligranulum variabile* was assigned as putative pectin cross-feeder based on its CAZyme carriage (Table 1), but as no growth on GalA or DP4 was detected, it is regarded as a background strain for co-culture experiments.

#### Maximum exponential growth rates of cultures exhibiting growth

For strains and substrates with a net OD_max_ >0.1, maximum exponential phase growth rates were determined (Tables 3 and S6). All *Bacteroidota* strains had relatively low growth rates on all substrates (0.14–0.64/h). The two *Lachnospira* species exhibited an extended lag phase (Figs 1 and S1) and similar growth rates on glucose (0.39/h and 0.44/h) and galacturonic acid (only *L. eligens* I42, 0.49/h), but growth on all backbone and pectin substrates was substantially faster (0.76–1.45/h). *Roseburia* pectin cross-feeders and *Faecalibacterium duncaniae* A2-165 had largely similar growth rates and grew well on DP4 oligo-galacturonan (0.77–1.0/h). *Faecalibacterium prausnitzii* SL3/3 generally exhibited lower growth rates (0.32–0.44/h). Mannose led to noticeably lower rates for *Roseburia* strains (Table S6), together with an extended lag phase (Fig. S1). Large growth rate differences were found between different background strains and substrates, with *Anaerostipes hadrus* SSC/2 growing the fastest of all strains (1.55/h on glucose, Table 3). *Anaerobutyricum hallii* DSM 3353 and *Ruminococcus bicirculans* 80/3 grew slowest (0.09/h on galactose and 0.24/h on glucose, respectively). Some strains grew fast on all substrates (for example, *Roseburia faecis* M72/1, 0.66–0.80/h), whereas others showed marked differences between substrates (for example, *E. rectale* A1-86, 0.23–0.92/h, Table S6).

### Growth of synthetic co-culture communities on different pectin-related substrates

#### Experimental setup

Eleven substrates were selected for synthetic co-culture studies, including commercial apple and citrus pectin, the five in-house pectins (apple, carrot, beetroot, beet leaf and kale), DP4 and DP5 oligo-galacturonans, a pectin monosaccharide mix consisting of the four major pectin monosaccharides of carrot pectin (65% galacturonic acid, 20.5% galactose, 10.5% arabinose and 3.5% rhamnose) and glucose. A total of 14 strains were selected, including 3 phylogenetically distant pectin degraders (designated P; *L. eligens* DSM 3376, *Segatella copri* DMS 18205 and *Phocaeicola* (formerly *Bacteroides*) *vulgatus* DSM 1447), two pectin cross-feeders (designated C; *Faecalibacterium duncaniae* A2-165 and *Roseburia intestinalis* M50/1) and all background strains (designated B and including *Subdoligranulum variabile* DSM 15176) apart from *Anaerobutyricum hallii* DSM 3353, based on its poor growth rate in pure culture (Table S6). All co-culture communities contained all background strains either with only cross-feeders (community BC), only pectin degraders (community BP) or both (community BCP; Table S3). In addition, BC communities were also incubated with only one of the pectin degraders (designated Le, Sc and Pv) on selected substrates. All strains were pre-grown on glucose (designated glc), and pectin degraders were also pre-grown on commercial apple pectin (designated pec) to assess if substrate adaptation affects their ability to compete in the community. All experiments were carried out in a single run, which enabled statistical comparison between different communities and substrates. The experiment was terminated after 19 h of incubation when the majority of the cultures had reached the stationary growth phase. The strain composition of all grown communities was assessed by multiplex PCR and fragment analysis. Relative abundances (%, Table S7) were used for statistical comparisons.

#### Co-culture growth characteristics

**Fig. 3.**
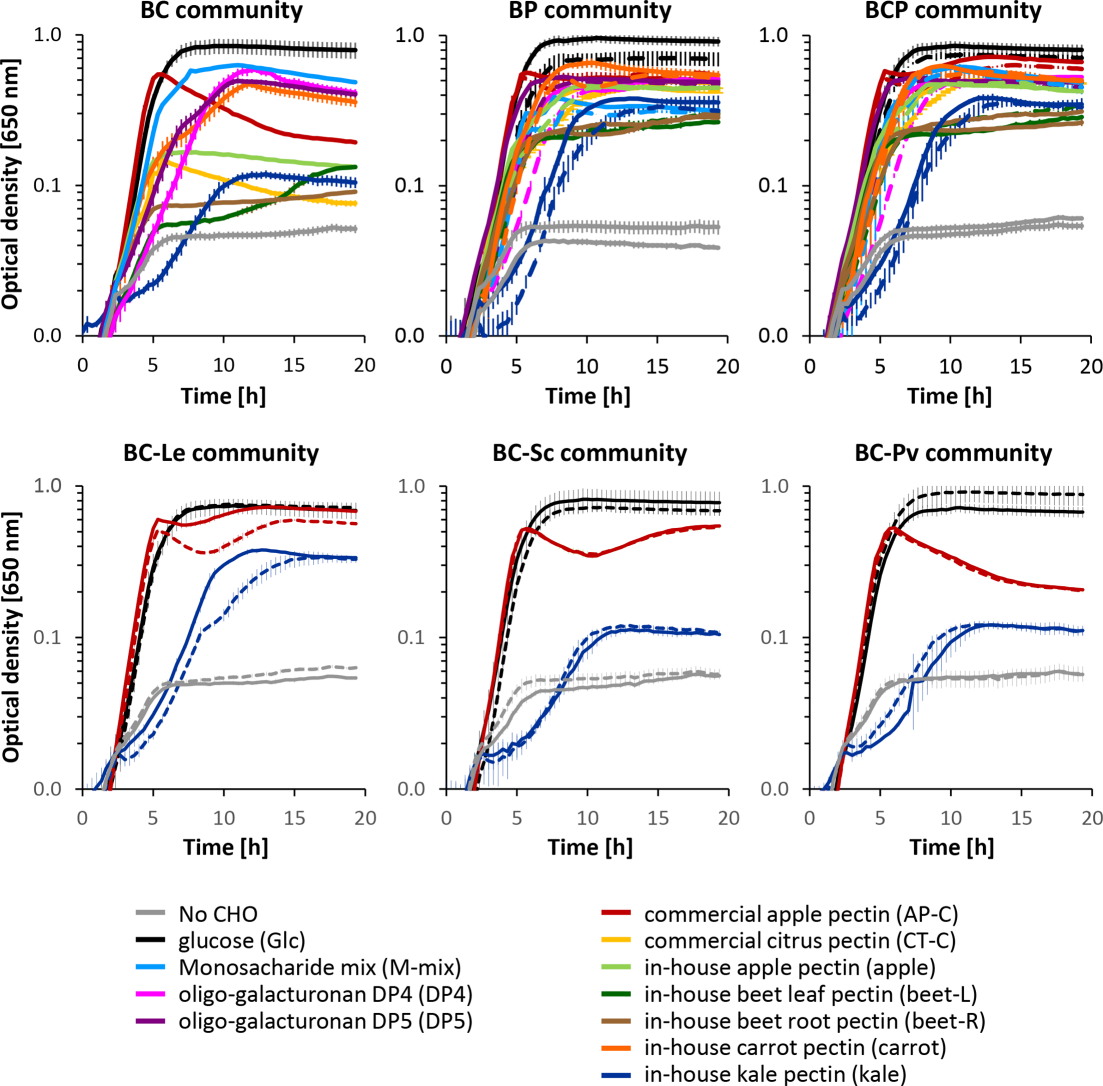
Co-culture growth of different microbial communities on different substrates and no CHO control. Community abbreviations: B, background strains; C, pectin cross-feeders; P, pectin degraders; Le, *L. eligens* DSM3376; Sc, *Segatella copri* DSM 18205; Pv, *P. vulgatus* DSM 1447; for strain details and growth data, see Table S3. Solid lines, pectin degraders were pre-grown on glucose; dashed lines, pectin degraders were pre-grown on commercial apple pectin. Monosaccharide-mix (M-mix): 65% galacturonic acid, 20.5% galactose, 10.5% arabinose and 3% rhamnose. Growth curves are means, and error bars show sd (*n*=3).

**Fig. 4.**
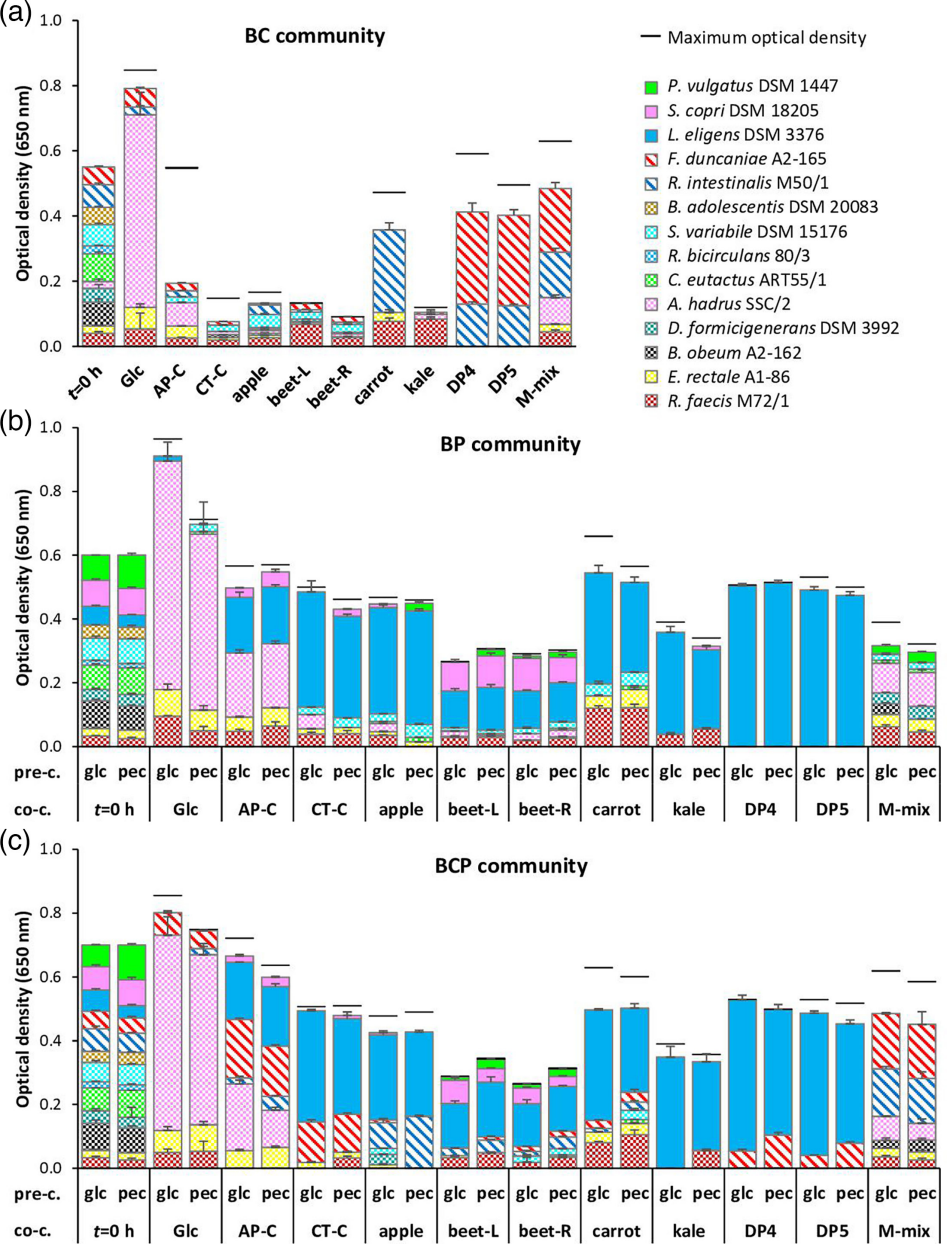
Microbial community composition on co-culture substrates (co-c.) glucose (Glc), commercial apple and citrus pectin (AP-C and CT-C), in-house apple, beet leaf (beet-L), beetroot (beet-R), carrot and kale pectin, oligo-galacturonan with four or five monomeric units (DP4 and DP5) and a monosaccharide mix resembling carrot pectin (65% galacturonic acid, 20.5% galactose, 10.5% arabinose and 3.5% rhamnose). (a) Background strains plus pectin cross-feeders (BC); (b) background strains plus pectin degraders (BP); (c) all 14 community strains (BCP) (for details, see Table S3). The three pectin degraders were pre-grown on either glucose (pre-c., glc) or commercial apple pectin (pre-c., pec). Average (*n*=3) and sd of relative abundance based on multiplex PCR and fragment analysis, scaled to the final OD at 19 h; black lines show the maximum OD. OD of inocula (*t*=0) is scaled to the number of strains times 0.05 OD equivalents.

There was little difference between the maximum and final OD, except for the BC and the BC-Pv communities incubated on commercial apple pectin (Fig. 3; growth data also provided in Table S3 and mapped onto Figs4  5). As expected, little growth (maximum OD <0.17) was observed for the BC community without pectin degraders on most pectins, except for carrot and commercial apple pectin, which reached a significantly (*P*<0.001) higher maximum OD (>0.45) compared to the other pectin sources (Fig. 3). When the pectin degraders were present (BP and BCP communities), there were generally higher levels of growth on all pectin sources, and both communities reached significantly (*P*<0.001) higher maximum and final OD on the commercial pectins, carrot and apple pectin, compared to the other pectin sources. The pre-culture substrate [glucose (glc) vs pectin (pec)] generally had little impact on community growth (Fig. 3). Furthermore, there were no significant differences in maximum or final OD between the BP and BCP communities grown on the same pectin source, indicating that the cross-feeders had little impact on community growth. In contrast, the BP community reached a significantly (*P*<0.001) lower maximum and final OD on the pectin-monosaccharide mix compared to the BC and BCP communities, indicating that the cross-feeders were important contributors to growth on this substrate.

**Fig. 5.**
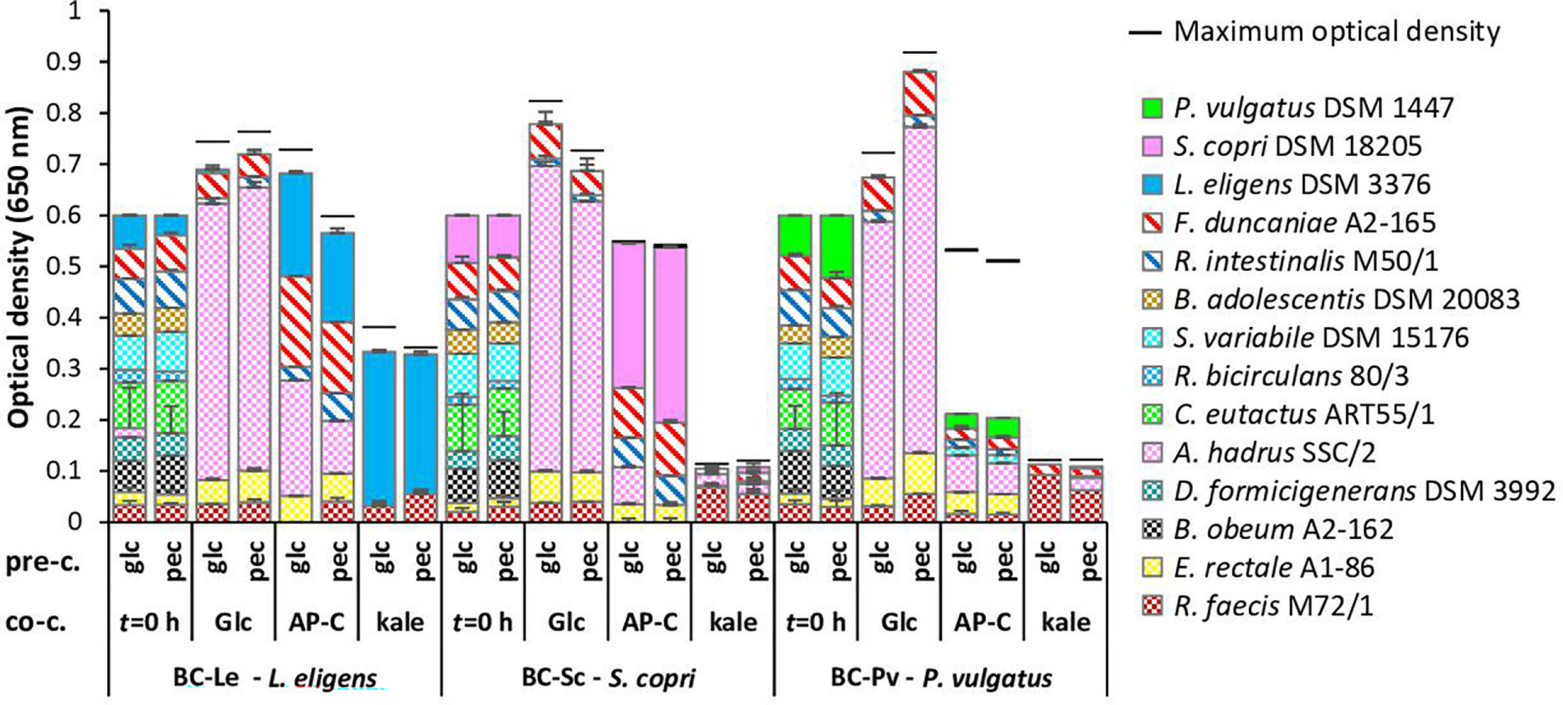
Microbial community composition on co-culture substrates (co-c.) glucose (Glc), commercial apple (AP-C) and kale pectin of background strains plus pectin cross-feeders (BC) with a single pectin degrader, *L. eligens* DSM 3376 (BC-Le), *Segatella copri* DSM 18205 (BC-Sc) and *P. vulgatus* DSM 1447 (BC-Pv) (for details, see Table S3). The three pectin degraders were pre-grown on either glucose (pre-c., glc) or commercial apple pectin (pre-c., pec). Average (*n*=3) and sd of relative abundance based on multiplex PCR and fragment analysis, scaled to the final OD at 19 h; black lines show the maximum OD. OD of inocula (*t*=0) is scaled to the number of strains times 0.05 OD equivalents.

The communities with single pectin degraders were grown on glucose, commercial apple pectin and kale pectin. Communities with *Segatella copri* DSM 18205 (BC-Sc) and *P. vulgatus* DSM 1447 (BC-Pv) displayed low growth on kale pectin (OD <0.13), regardless of the pre-culture substrate (Fig. 3). In contrast, the communities with *L. eligens* DSM 3376 (BC-Le) did grow on kale pectin and reached a similar final OD to the BCP community incubated on kale (final OD 0.35–0.38; albeit with a lower growth trajectory for the pectin-grown pre-culture, Fig. 3). Communities with *L. eligens* DSM 3376 (BC-Le) and *Segatella copri* DSM 18205 (BC-Sc), but not *P. vulgatus* (BC-Pv), as a sole pectin degrader grew well on commercial apple pectin, irrespective of the pre-culture substrate (Fig. 3).

#### Effect of pre-culture substrate of pectin degraders on microbial community composition

There were no major differences in community composition between co-cultures that had been inoculated with pectin degraders grown on either glucose or commercial apple pectin (Figs4  5, % data scaled to overall growth based on final OD). Permutational ANOVA (PERMANOVA) of the Bray–Curtis beta-diversity suggested that an insignificant amount of the variation found in BP and BCP community composition (0.5% and 0.8%, *P*=0.967 and 0.941, respectively) could be explained by the pre-culture substrate, and it also did not affect the hierarchical order between pectin degraders. The main drivers of differences in community composition were the substrates, explaining 57.4% of the variation (*P*<0.001, PERMANOVA), and the inocula (BC, BP and BCP), explaining 25.4% (*P*<0.001). The focus in further sections will therefore be on the communities with glucose as a pre-culture substrate.

#### Microbial composition of BC, BP and BCP communities

Glucose-grown communities were dominated by *Anaerostipes hadrus* SSC/2 (74–79%), in accordance with its high pure culture growth rate (Table 3). *E. rectale* A1-86 and *Roseburia faecis* M72/1 were also present and often detectable on other substrates as well (Fig. 4). Pectin cross-feeders *Faecalibacterium duncaniae* A2-165 and *Roseburia intestinalis* M50/1 (BC and BCP communities) also became detectable in most glucose-grown cultures and were the dominant strains on the pectin monosaccharide mix, in accordance with its high galacturonic acid content. Furthermore, they were the only strains detected on both oligo-galacturonans in the absence of pectin degraders (BC community), and *Roseburia intestinalis* M50/1 was the most dominant strain on carrot pectin in this community (71%). In contrast, *Faecalibacterium duncaniae* A2-165 was not detected on carrot pectin in the BC community but reached a significantly (*P*<0.001) higher relative abundance on the oligo-galacturonans (69%) and the pectin monosaccharide mix (40%), compared to the pectin substrates (Fig. 4). In the presence of the pectin degraders (BCP community), *Faecalibacterium duncaniae* A2-165 showed a significant increase (*P*<0.001) in relative abundance on the HG-rich commercial pectins compared to the BC community and outcompeted *Roseburia intestinalis* M50/1. Similarly for the oligo-galacturonans, *Faecalibacterium duncaniae* A2-165 was the only detectable cross-feeder in the presence of pectin degraders. In contrast, *Roseburia intestinalis* M50/1 reached a significantly (*P*<0.001) higher relative abundance (19%) on the in-house apple pectin compared to the other in-house pectins in the presence of pectin degraders (Fig. 4, BCP community).

*Bautia obeum* A2-162, *Coprococcus eutactus* ART55/1, *Dorea formicigenerans* DSM 3392, *Ruminococcus bicirculans* 80/3 and *Bifidobacterium adolescentis* DSM 20083 did not compete well on any of the substrates, but *Subdoligranulum variabile* DSM 15176 was detectable on some pectin substrates (Fig. 4). Comparison of the BC community to the communities with pectin degraders (BP and BCP) generally showed little evidence of cross-feeding to background strains. The lactate utilizer *Anaerostipes hadrus* SSC/2, however, showed a significantly (*P*<0.001) higher relative abundance when grown on commercial apple pectin together with the pectin degraders than in their absence, which may be due to lactate cross-feeding. *L. eligens* DSM 3376 was the dominant pectin degrader (BP and BCP communities), and the introduction of cross-feeders generally had little impact on its abundance, except for significantly lower relative abundance on apple pectin (*P*<0.001) and the oligo-galacturonans (*P*<0.05) (Fig. 4). *Segatella copri* DSM 18205 reached a significantly (*P*<0.001) higher relative abundance on the arabinose-rich beet pectins compared to the other substrates (BP and BCP communities), but it showed a significant (*P*<0.001) decrease in relative abundance when the cross-feeders were introduced (Fig. 4), suggesting competition. *P. vulgatus* DSM 1447 did not compete well on any of the substrates and reached detectable levels mainly on the beet pectins, as well as on the pectin monosaccharide mix in the absence of cross-feeders (Fig. 4).

#### Microbial composition of communities with single pectin degraders

All glucose-grown communities displayed a similar composition as seen with the BCP community for communities with single pectin degraders, as were *L. eligens* DSM 3376-containing communities (BC-Le) on commercial apple and kale pectin (Fig. 5). In the BC-Sc community, *Segatella copri* DSM 18205 was the dominant strain after incubation on commercial apple pectin. Low growth (final OD <0.15) was seen on kale pectin for the communities with either *Segatella copri* DSM 18205 (BC-Sc) or *P. vulgatus* DSM 1447 (BP-Pv) as sole pectin degrader. For BC-Pv, neither the pectin degrader nor the pectin cross-feeders grew well (Fig. 5), although due to a large drop in OD it cannot be excluded that cell lysis had occurred by the time samples were taken at the end of the growth experiment (Fig. 3). On commercial apple pectin, *L. eligens* DSM 3376 had a lower relative abundance (BC-Le communities) than *Segatella copri* DSM 18205 (BC-Sc communities), suggesting that it enabled more cross-feeding to other community members, in particular *Anaerostipes hadrus* SSC/2 and *Faecalibacterium duncaniae* A2-165 (Fig. 5).

## Discussion

Pectin has been warranted a health claim by the European Food Safety Authority (EFSA) based on its positive impact on blood glucose due to its gelling properties, which slow the absorption of glucose in the small intestine [22]. It has also been suggested to strengthen the gastrointestinal immune barrier by interacting with pattern recognition receptors [23]. The stimulation of certain gut microbes may also contribute to the health effects of pectins. For example, the pectin degrader *L. eligens* DSM 3376 has been shown *in vitro* to induce the production of the anti-inflammatory cytokine IL-10 [7], and a patent has been granted for the use of this species in the treatment of colitis or colorectal cancer [24]. The genus *Faecalibacterium* consists of butyrate producers, and its reduced abundance has been linked to intestinal disorders such as ulcerative colitis and Crohn’s disease [25]. Here, we confirmed that the utilization of intact pectin is limited among human gut bacteria. In co-culture, *L. eligens* DSM 3376 was the dominant pectin degrader on all tested pectin sources, in accordance with its higher growth rates in pure culture on most pectins compared to *Bacteroidota* strains. It grew poorly on beet pectins in pure culture, which was reflected in other pectin degraders, and in particular *Segatella copri* DSM 18205, competing better in co-culture on those pectins. The 96-well culture system used here may disadvantage the *Bacteroidota* strains; however, efficient competition of bacteria belonging to *L. eligens* with *Bacteroides* species on commercial apple pectin has also been shown in continuous human faecal microbiota continuous culture fermenters [26]. In that study, *Bacteroides* species competed better at higher pH values. The 96-well system utilized here does not allow for pH control; therefore, decreasing pH during fermentation may have benefitted *L. eligens* DSM 3376.

*Segatella copri* DSM 18205 displayed an increased ability to compete on the arabinose-rich beet pectins [11] and was the only strain growing on arabinan in pure culture. Furthermore, bacteria belonging to *Segatella copri* have previously been positively associated with arabinose-rich pectin [10]. When grown together, it is therefore likely that *Segatella copri* and *L. eligens* consume different parts of the beet pectin molecule. The introduction of the pectin cross-feeders resulted in a significantly lower relative abundance of *Segatella copri* DSM 18205 after growth on the beet pectins, suggesting competition for pectin breakdown products. In contrast, as the sole pectin degrader on the HG-rich commercial apple pectin, it may operate as a primary pectin degrader. Adapting to the presence of other strains has been observed in other bacteria, for example, by transport protein regulation [27]. A preference for a certain part of the pectin molecule has also been demonstrated for *Bacteroides* species [28], as well as a hierarchical order of preference for different carbohydrates [29]. *Bacteroides* species regulate CAZyme gene expression in response to substrate availability [30]. In contrast, *L. eligens* DSM 3376 expresses its major pectin lyase (PL9) constitutively [7], similar to the constitutive expression of amylases in the highly specialized starch degrader *Ruminococcus bromii* [31]. Pre-cultures of the pectin degraders were grown on both glucose and pectin to assess if gene regulation may provide a competitive advantage to *Bacteroidota* strains. However, growth on pectin did not increase the relative abundance of *P. vulgatus* DSM 1447 or *Segatella copri* DSM 18205 in co-culture with *L. eligens* DSM 3376.

Our work suggests that only strains with pectin-specific PLs can utilize intact pectin. *Faecalibacterium duncaniae* A2-165 grew on DP4 oligo-galacturonan and galacturonic acid in pure culture, but the level of growth in co-culture depended on the substrate. In the presence of pectin degraders, only the HG-rich commercial pectins resulted in a high relative abundance of *Faecalibacterium duncaniae* A2-165. The two pectin cross-feeders have relatively similar CAZyme profiles for the breakdown of HG, but they differ greatly in their profiles regarding the breakdown of the RG-I side chains, with *Roseburia intestinalis* M50/1 possessing a greater number of GH-encoding genes and more GH families. *Faecalibacterium duncaniae* A2-165 therefore appears to be more specialized to HG cross-feeding, whereas *Roseburia intestinalis* M50/1 may utilize both HG and the RG-I side chains, which is in agreement with it competing better on some of the more RG-I side chain-rich in-house pectins. An *in vitro* faecal fermentation study showed a positive correlation of *Faecalibacterium* with galacturonic acid, and its decreased relative abundance with RG-I as a growth substrate, which supports its potential specialization towards cross-feeding of HG [10]. In this study, there was generally little cross-feeding to the background community, demonstrating a rather ‘selfish’ behaviour of the pectin degraders and cross-feeders. The lactate utilizer *Anaerostipes hadrus* SSC/2 competed well in co-cultures on commercial apple pectin. As it also displayed some growth on this substrate in pure culture, it is not clear if this observation was due to lactate cross-feeding.

Synthetic co-culture studies allow for the assessment of microbial interactions in a completely defined system, but they are naturally limited by the number of strains that can be included and therefore much less complex than the human gut microbiota. There might be other important species in pectin metabolism that were not investigated here, and there can be functional variation between closely related species [13,32] and even between strains of a given species, as found here and elsewhere [33]. One interesting observation from this study that highlights the potential importance of inter-individual microbiota variation for pectin fermentation in the gut was the strain differences observed between the two *E. eligens* strains. *E. eligens* DSM 3376 did not grow well on galacturonic acid and the beet pectins, which was reflected in its inferior competition on those pectins in co-culture. *E. eligens* I42, on the other hand, grew well on those substrates. Another limitation of this study is the inability to regulate the pH in small-scale batch cultures, with pH changes affecting different community members to a different degree [34]. Nevertheless, they allow the parallel assessment of more microbial communities and substrates than possible in more complex model systems and enable the generation of hypotheses that can be followed up in systems that model the human intestine more closely or enable further analyses, such as metatranscriptomic analysis, as demonstrated by Shetty *et al*. [12,35]. Furthermore, the underlying biochemistry of pectin metabolism in the main microbial players with regard to enzyme kinetics and carbohydrate transporters, together with the assessment of changes in pectin structure during microbial utilization of pure cultures and microbial communities, is necessary to gain a more complete understanding of pectin metabolism by the human gut microbiota.

## Conclusion

This work demonstrates that *L. eligens* DSM 3376 is a highly specialized pectin degrader, evidenced by its very narrow substrate growth spectrum, together with very high growth rates on oligo- and polysaccharides compared to monosaccharides. It outcompeted the two other pectin degraders on a variety of pectin sources. Although *P. vulgatus* DSM 1447 and *Segatella copri* DSM 18205 can utilize pectin in pure culture, they appear to act more as cross-feeders in the synthetic co-culture communities under the conditions employed here. The effectiveness of two cross-feeders (*Faecalibacterium duncaniae* A2-165 and *Roseburia intestinalis* M50/1) to utilize pectin breakdown products was dependent on the pectin source, which suggests that pectin composition is an important factor in cross-feeding interactions. This is in line with a diverse whole plant-based diet supporting a diverse pectin-degrading community. Our results will help to explain the consequences of inter-individual variation, such as differences in pectin metabolism between individuals with *Bacteroides*- or *Prevotella*-dominant microbiota composition. Furthermore, they will contribute to the rational development of dietary strategies for personalized nutrition approaches (tailored nutrition advice or targeted novel dietary supplements) to promote beneficial microbes.

## Supplementary material

10.1099/mic.0.001559 Uncited Supplementary Material 1.

10.1099/mic.0.001559 Uncited Supplementary Material 2.

## Abbreviations

AIR: alcohol-insoluble residue
CAZyme: carbohydrate-active enzyme
CE: carbohydrate esterase
CHO: carbohydrate
DP: degree of polymerization
GH: glycoside hydrolase
HG: homogalacturonan
PERMANOVA: permutational ANOVA
PL: polysaccharide lyase
RFU: relative fluorescence unit
RG-I: rhamnogalacturonan I
RG-II: rhamnogalacturonan II
YCFA: yeast extract casitone fatty acids
