# Supplementary material for: Roles of human colonic bacteria in pectin utilization and associated cross-feeding networks revealed using synthetic co-cultures
Source: Microbiology (Reading). 2025 May 29;171(5):001559. doi: 10.1099/mic.0.001559 (PMC12163728; doi:10.1099/mic.0.001559)
Supplement: Uncited Supplementary Material 1. [file mic-171-01559-s001.pdf]

Fig. S1. Pure culture growth curves for 22 bacterial strains. Growth curves removed from analysis due to faulty data are not shown here, but all growth data are provided in Table S4. Data are shown on a linear scale to facilitate optimal visualisation of lag phases and final optical densities. Maximum growth rates (Table S6) were determined from logarithmic curves (for details see Table S4).

Substrate abbreviations:
Monosaccharides: Ara, arabinose; Gal, galactose; GalA, galacturonic acid; Glc, glucose; Man, mannose; Rha, rhamnose; Xyl, xylose.
RG-I side chain & backbone carbohydrates: AB, arabinan; ABG, arabinogalactan; Gal-AN, galactan; DP4/DP6/DP8, oligo-galacturonan with four/six/eight monomeric units; RG, rhamnogalacturonan; HG, homogalacturonan.
Pectins: AP-C/CT-C, commercial apple/citrus pectin; apple/beet-L/beet-R/carrot/kale, in-house apple/beet leaf/beetroot/carrot/kale pectin.

p. 1-2: Overview of strains and substrates utilised in five pure culture growth experiments (E1-E5).
p. 3-4: All strains – growth on glucose and no CHO control.
p. 5-6: All strains – growth on monosaccharides.
p. 7-8: All strains – growth on oligo- and polysaccharides.
p. 9: Pectin degraders and cross feeders – growth on oligo-galacturonans and homogalacturonan.
p. 10: Pectin degraders – growth on pectins.

	Monosaccharides																								
	Glc					Ara			Gal			GalA					Man			Rha			Xyl		
B. ovatus V975		E2	E3				E2			E2			E2	E3				E2			E2			E2	
B. thetaiotaomicron VPI-5482		E2	E3	E4			E2			E2	E4		E2	E3				E2			E2	E4		E2	
P. vulgatus DSM 1447		E2	E3				E2			E2			E2	E3				E2			E2			E2	
S. copri DSM 18205		E2	E3				E2			E2			E2	E3				E2			E2			E2	
L. eligens DSM 3376		E2	E3		E5		E2			E2			E2	E3		E5		E2			E2			E2	
L. eligens I42		E2	E3	E4	E5		E2			E2	E4		E2	E3	E4	E5		E2			E2			E2	
F. duncaniae A2-165	E1		E3	E4		E1			E1		E4	E1		E3			E1			E1			E1		
F. prausnitzii SL3/3	E1		E3	E4		E1			E1		E4	E1		E3			E1			E1			E1		E4
R. hominis A2-183	E1		E3			E1			E1			E1		E3			E1			E1			E1		
R. intestinalis L1-82				E4	E5			E4			E4				E4				E4			E4			E4
R. intestinalis M50/1	E1		E3		E5	E1			E1			E1		E3			E1			E1			E1		
R. intestinalis XB6B4			E3	E4	E5			E4			E4			E3					E4			E4			E4
S. variabile DSM 15176	E1			E4		E1		E4	E1		E4	E1					E1			E1			E1		
R. faecis M72/1	E1			E4	E5	E1			E1		E4	E1					E1			E1			E1		
R. rectale A1-86	E1			E4		E1			E1		E4	E1					E1			E1			E1		
A. hadrus SSC/2	E1			E4		E1			E1		E4	E1					E1			E1			E1		
A. hallii DSM 3353	E1			E4		E1			E1		E4	E1					E1			E1			E1		
B. obeum A2-162	E1			E4		E1			E1		E4	E1					E1			E1			E1		
C. eutactus ART55/1	E1			E4		E1			E1		E4	E1					E1			E1			E1		
D. formicigenerans DSM 3992	E1			E4		E1			E1		E4	E1					E1			E1			E1		
R. bicirculans 80/3	E1			E4		E1		E4	E1		E4	E1					E1			E1			E1		
B. adolescentis DSM 20083	E1			E4		E1			E1		E4	E1					E1			E1			E1		E4

	RG-I side chain & backbone carbohydrates																					
	AB			ABG			Gal-AN			DP4				DP6			DP8		RG			HG
B. ovatus V975		E3			E3		E3				E2	E3		E3			E3			E3		E3
B. thetaiotaomicron VPI-5482		E3			E3		E3	E4			E2	E3		E3			E3			E3		E3
P. vulgatus DSM 1447		E3			E3		E3				E2	E3		E3			E3			E3		E3
S. copri DSM 18205		E3			E3		E3				E2	E3		E3			E3			E3		E3
L. eligens DSM 3376		E3			E3		E3				E2	E3		E3			E3			E3		E3
L. eligens I42		E3	E4		E3	E4	E3	E4			E2	E3	E4	E3	E4		E3	E4		E3	E4	E3
F. duncaniae A2-165	E1	E3		E1	E3		E3	E4		E1		E3		E3			E3		E1	E3		E3
F. prausnitzii SL3/3	E1	E3		E1	E3		E3	E4		E1		E3	E4	E3			E3		E1	E3		E3
R. hominis A2-183	E1	E3		E1	E3		E3			E1		E3		E3			E3		E1	E3		E3
R. intestinalis L1-82			E4			E4		E4	E5				E4		E4	E5		E4			E4	E4
R. intestinalis M50/1	E1	E3		E1	E3		E3		E5	E1		E3		E3		E5	E3		E1	E3		E3
R. intestinalis XB6B4		E3			E3		E3	E4	E5			E3		E3		E5	E3			E3		E3
S. variabile DSM 15176	E1			E1				E4		E1									E1			
R. faecis M72/1	E1			E1		E4		E4	E5	E1									E1			
R. rectale A1-86	E1			E1				E4		E1									E1			
A. hadrus SSC/2	E1			E1				E4		E1									E1			
A. hallii DSM 3353	E1			E1				E4		E1									E1			
B. obeum A2-162	E1			E1				E4		E1									E1			
C. eutactus ART55/1	E1			E1				E4		E1									E1			
D. formicigenerans DSM 3992	E1			E1				E4		E1									E1			
R. bicirculans 80/3	E1			E1				E4		E1									E1			
B. adolescentis DSM 20083	E1			E1				E4		E1									E1			

	pectins												
	AP-C				CT-C	apple		b.-L*	b.-R*	carrot			kale
B. ovatus V975		E2			E2	E2	E3	E2	E2		E2		E2
B. thetaiotaomicron VPI-5482		E2			E2	E2	E3	E2	E2		E2		E2
P. vulgatus DSM 1447		E2			E2	E2	E3	E2	E2		E2		E2
S. copri DSM 18205		E2			E2	E2	E3	E2	E2		E2		E2
L. eligens DSM 3376		E2		E5	E2	E2	E3	E2	E2		E2		E2
L. eligens I42		E2		E5	E2	E2	E3	E2	E2		E2		E2
F. duncaniae A2-165	E1						E3			E1			
F. prausnitzii SL3/3	E1						E3			E1			
R. hominis A2-183	E1						E3			E1			
R. intestinalis L1-82			E4									E4	
R. intestinalis M50/1	E1						E3			E1			
R. intestinalis XB6B4			E4				E3					E4	
S. variabile DSM 15176	E1									E1			
R. faecis M72/1	E1									E1			
R. rectale A1-86	E1									E1			
A. hadrus SSC/2	E1									E1			
A. hallii DSM 3353	E1									E1			
B. obeum A2-162	E1									E1			
C. eutactus ART55/1	E1									E1			
D. formicigenerans DSM 3992	E1									E1			
R. bicirculans 80/3	E1									E1			
B. adolescentis DSM 20083	E1									E1			

*b.-L, beet leaf; b.-R, beetroot

All strains - Glucose and no CHO control

legend see p. 4

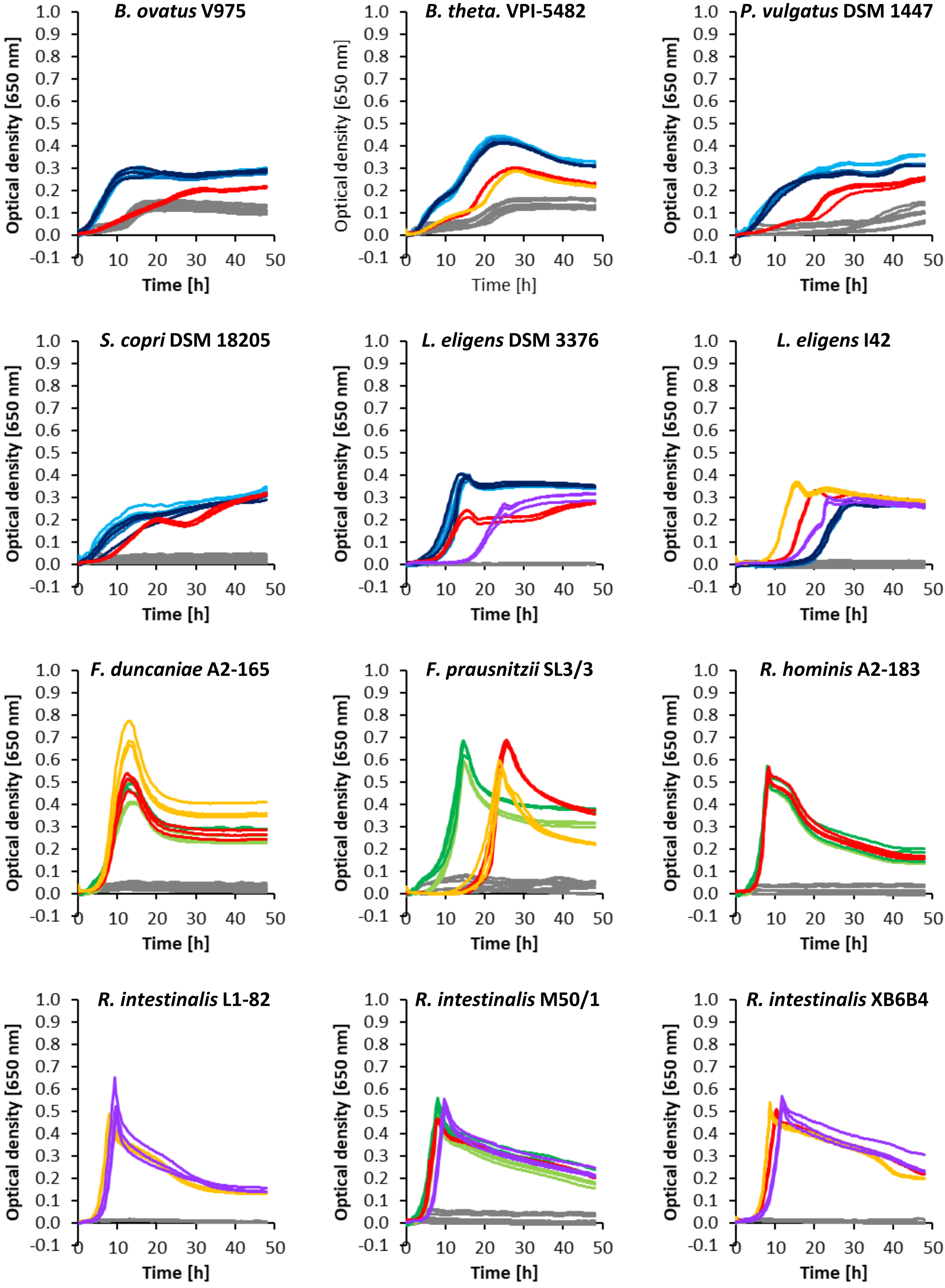

All strains - Glucose and no CHO control

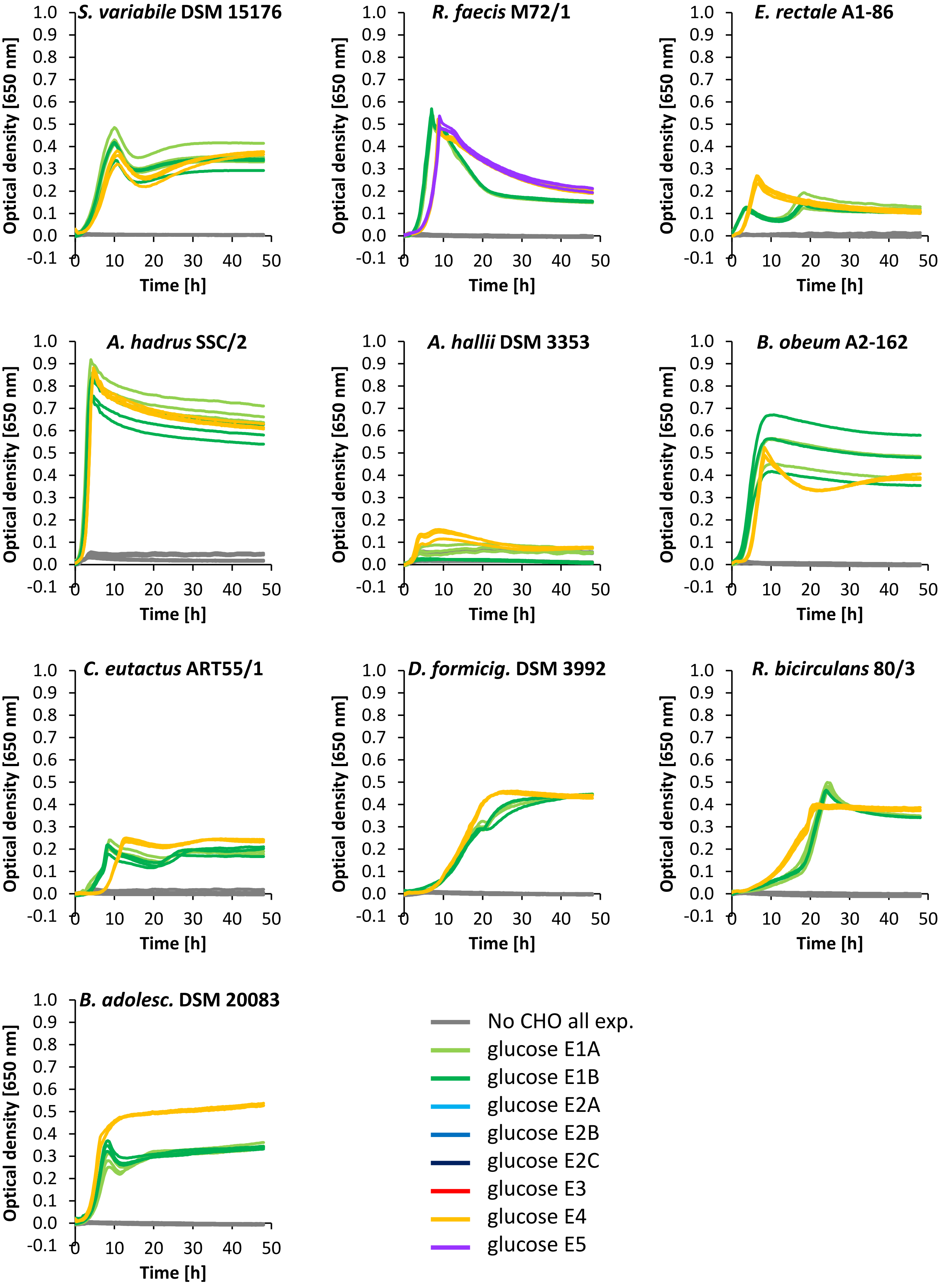

All strains - Monosaccharides

legend see p. 6

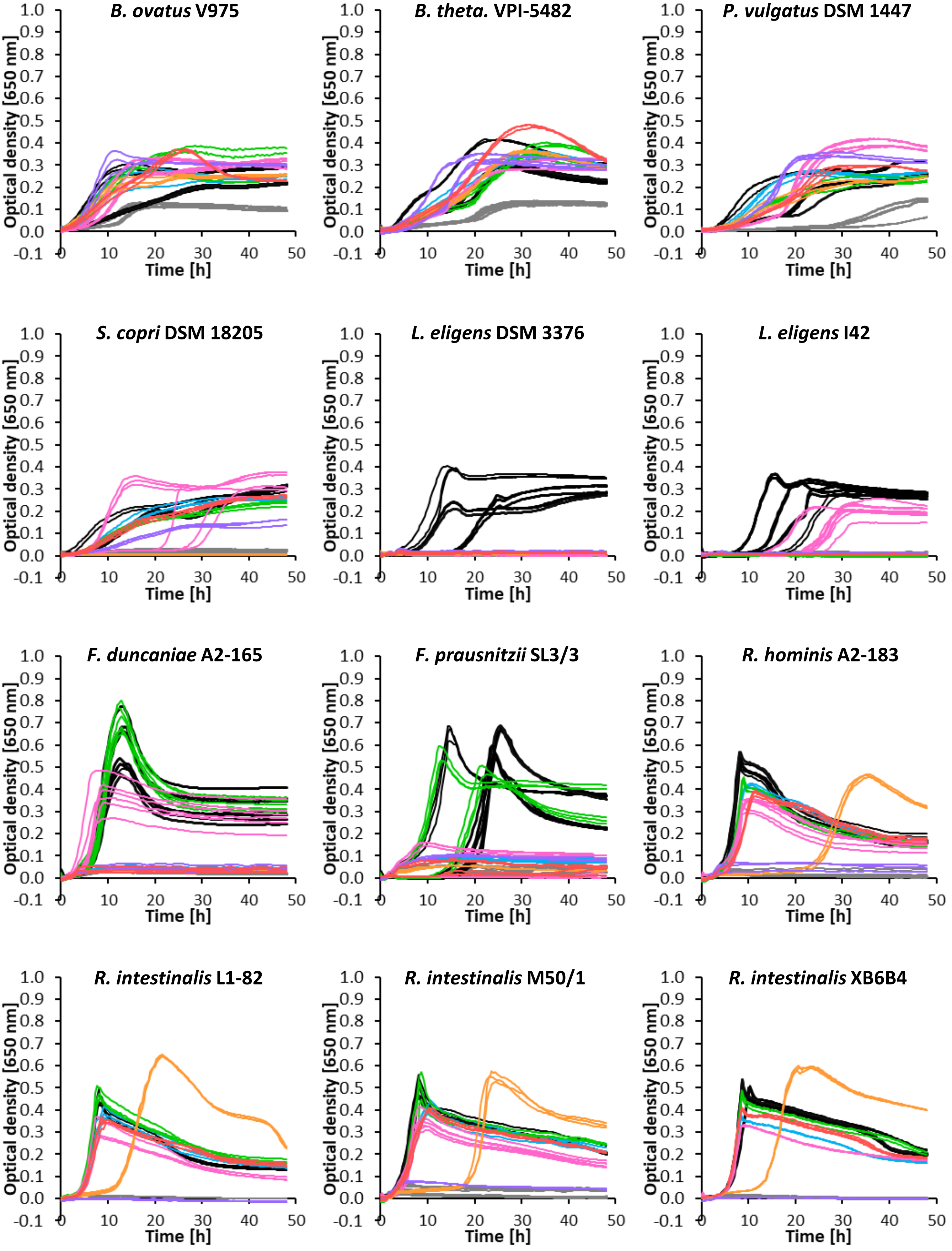

All strains - Monosaccharides

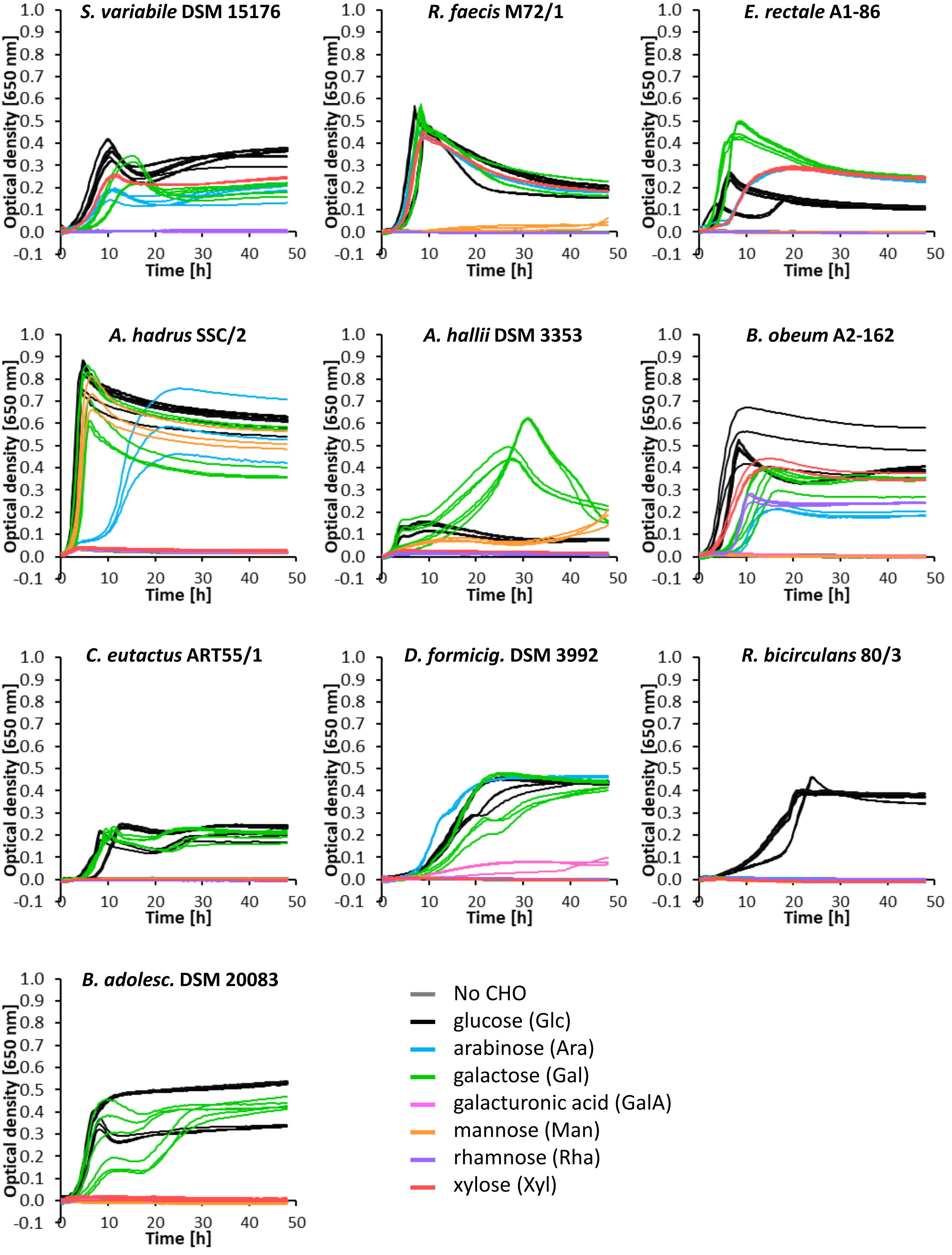

All strains - Oligo- and polysaccharides

legend see p. 8

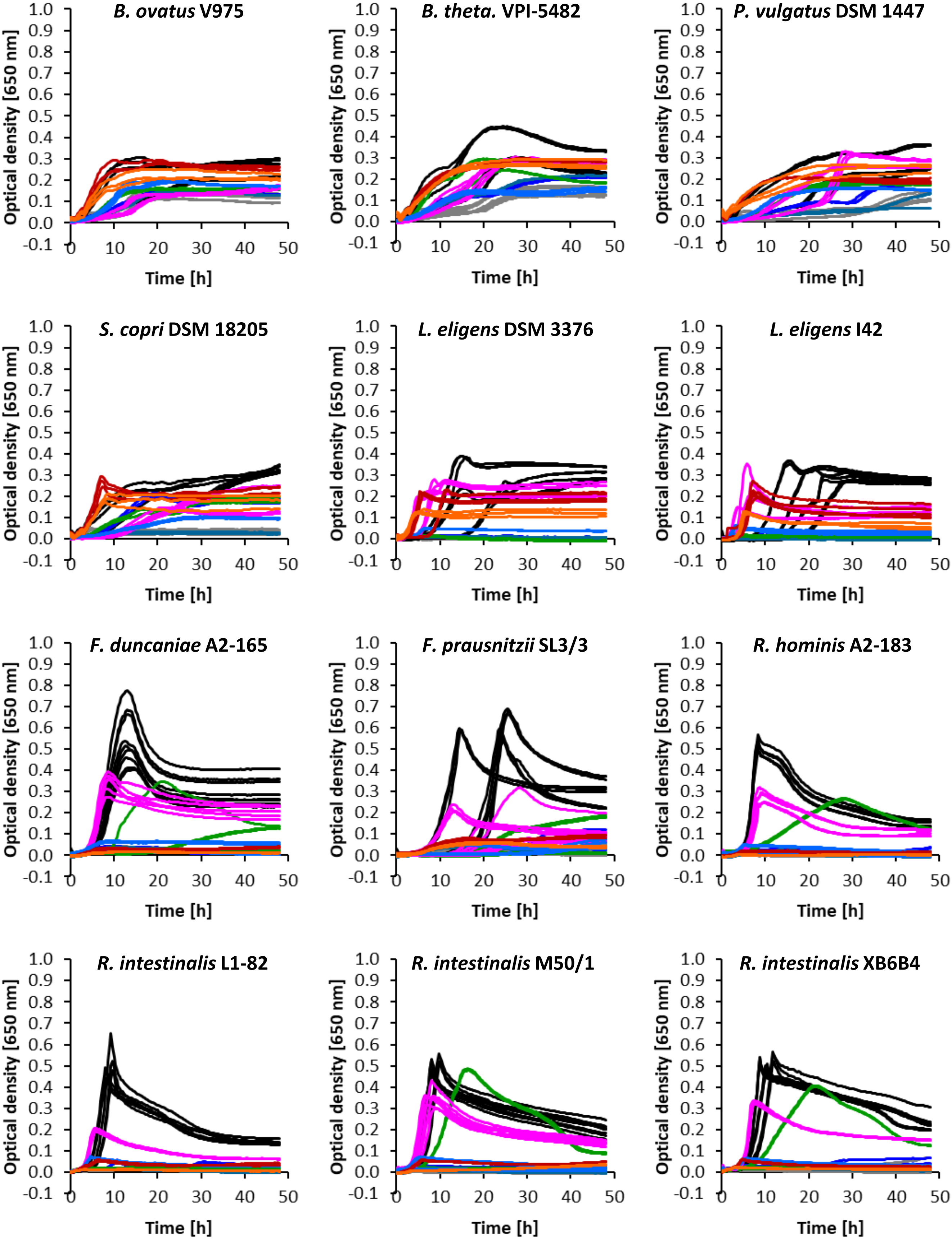

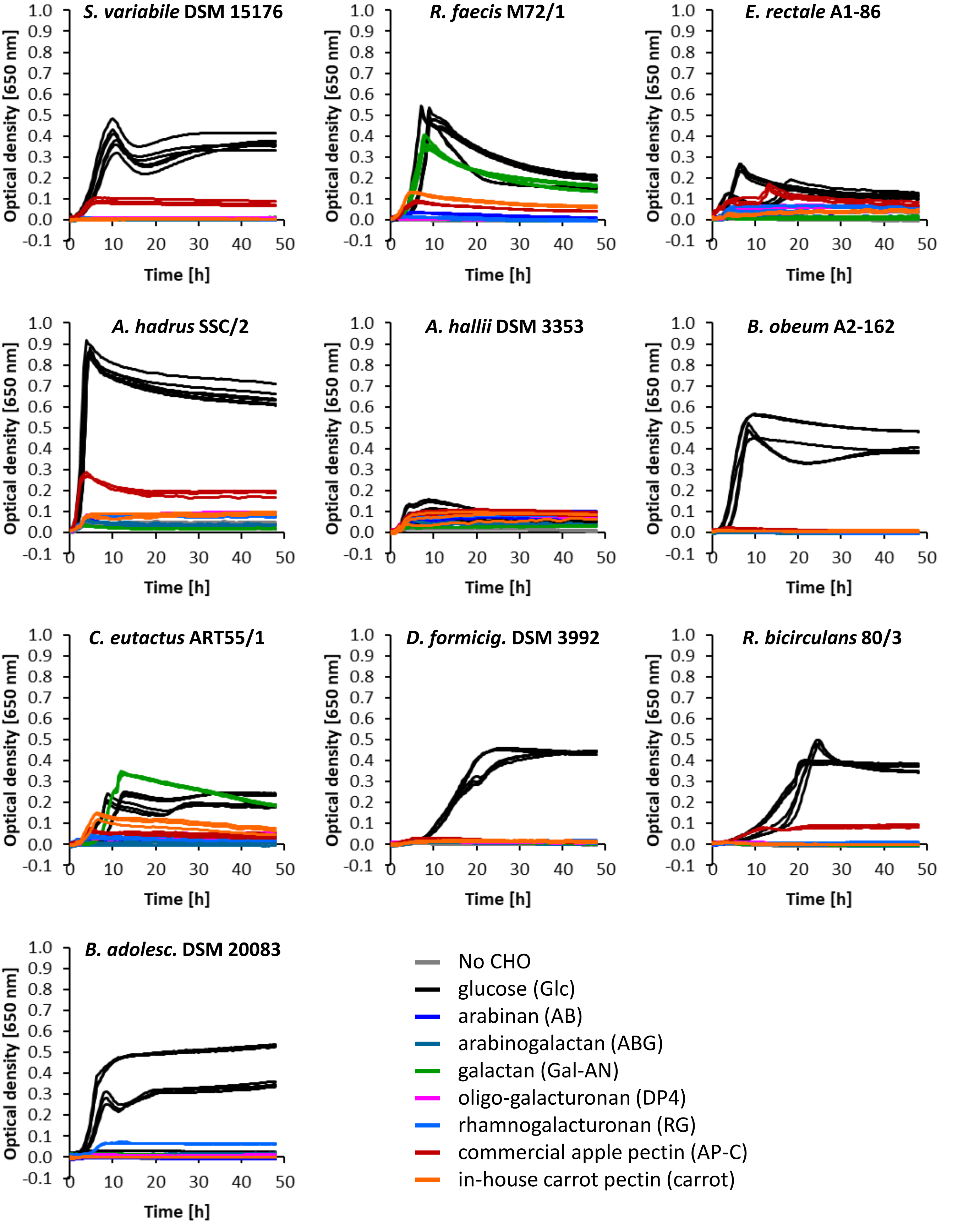
S. variabile DSM 15176
R. faecis M72/1
E. rectale A1-86
A. hadrus SSC/2
A. hallii DSM 3353
B. obeum A2-162
C. eutactus ART55/1
D. formicig. DSM 3992
R. bicirculans 80/3
B. adolesc. DSM 20083
Optical density [650 nm]
Time [h]
1.0 0.9 0.8 0.7 0.6 0.5 0.4 0.3 0.2 0.1 0.0 -0.1
0 10 20 30 40 50
No CHO
glucose (Glc)
arabinan (AB)
arabinogalactan (ABG)
galactan (Gal-AN)
oligo-galacturonan (DP4)
rhamnogalacturonan (RG)
commercial apple pectin (AP-C)
in-house carrot pectin (carrot)

Pectin degraders and cross feeders – oligo-galacturonans (DP4, DP6, DP8) & homogalacturonan

legend see p. 10

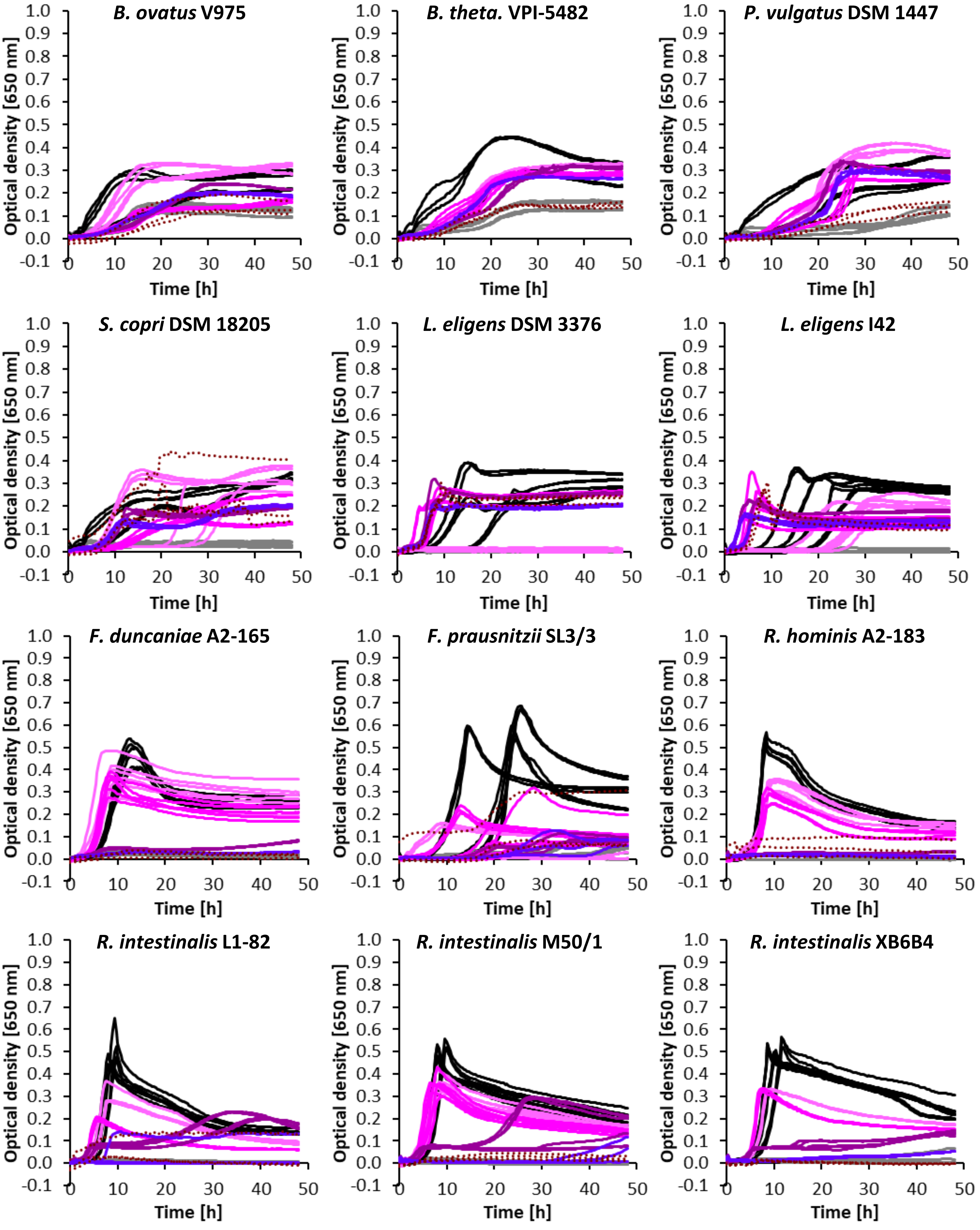

Pectin degraders – pectins

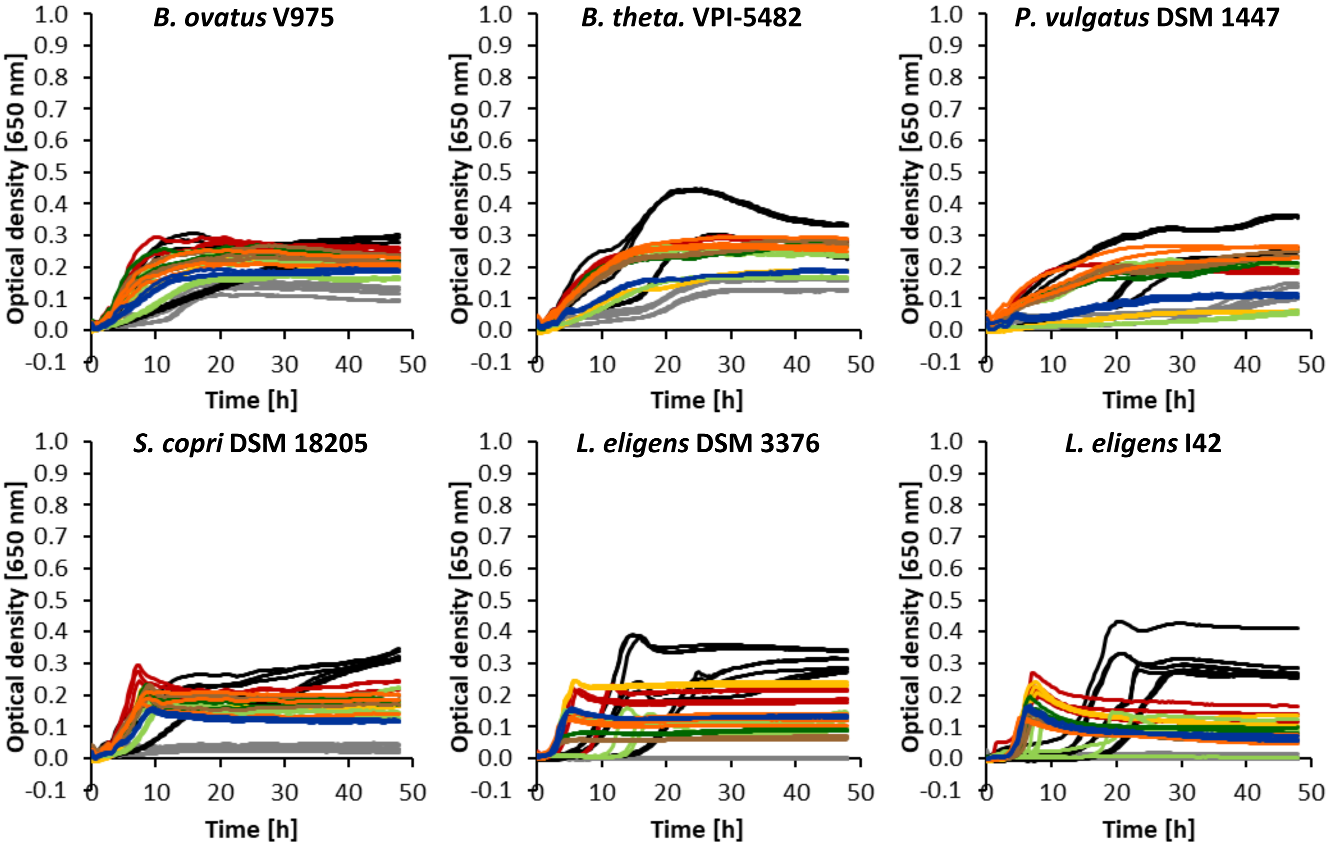

p. 9

- No CHO all exp.
- glucose (Glc)
- galacturonic acid (GalA)
- oligo-galacturonan DP4
- oligo-galacturonan DP6
- oligo-galacturonan DP8
- homogalacturonan (HG)

p. 10

- commercial apple pectin (AP-C)
- commercial citrus pectin (CT-C)
- in-house apple pectin (apple)
- in-house beet leaf pectin (beet-L)
- in-house beet root pectin (beet-R)
- in-house carrot pectin (carrot)
- in-house kale pectin (kale)
